# The cost-effectiveness of alternative vaccination strategies for polyvalent meningococcal vaccines in Burkina Faso: A transmission dynamic modeling study

**DOI:** 10.1371/journal.pmed.1002495

**Published:** 2018-01-24

**Authors:** Reza Yaesoubi, Caroline Trotter, Caroline Colijn, Maziar Yaesoubi, Anaïs Colombini, Stephen Resch, Paul A. Kristiansen, F. Marc LaForce, Ted Cohen

**Affiliations:** 1 Department of Health Policy and Management, Yale School of Public Health, New Haven, Connecticut, United States of America; 2 Department of Veterinary Medicine, University of Cambridge, Cambridge, United Kingdom; 3 Department of Mathematics, Imperial College London, London, United Kingdom; 4 Centre for Mathematics of Precision Healthcare, Imperial College London, London, United Kingdom; 5 Department of Electrical and Computer Engineering, University of New Mexico, Albuquerque, New Mexico, United States of America; 6 Gavi, Geneva, Switzerland; 7 Department of Health Policy and Management, Harvard School of Public Health, Boston, Massachusetts, United States of America; 8 Department of Bacteriology, Norwegian Institute of Public Health, Oslo, Norway; 9 Serum Institute of India, Pune, India; 10 Department of Epidemiology of Microbial Diseases, Yale School of Public Health, New Haven, Connecticut, United States of America; Mahidol-Oxford Tropical Medicine Research Unit, THAILAND

## Abstract

**Background:**

The introduction of a conjugate vaccine for serogroup A *Neisseria meningitidis* has dramatically reduced disease in the African meningitis belt. In this context, important questions remain about the performance of different vaccine policies that target remaining serogroups. Here, we estimate the health impact and cost associated with several alternative vaccination policies in Burkina Faso.

**Methods and findings:**

We developed and calibrated a mathematical model of meningococcal transmission to project the disability-adjusted life years (DALYs) averted and costs associated with the current Base policy (serogroup A conjugate vaccination at 9 months, as part of the Expanded Program on Immunization [EPI], plus district-specific reactive vaccination campaigns using polyvalent meningococcal polysaccharide [PMP] vaccine in response to outbreaks) and three alternative policies: (1) Base Prime: novel polyvalent meningococcal conjugate (PMC) vaccine replaces the serogroup A conjugate in EPI and is also used in reactive campaigns; (2) Prevention 1: PMC used in EPI and in a nationwide catch-up campaign for 1–18-year-olds; and (3) Prevention 2: Prevention 1, except the nationwide campaign includes individuals up to 29 years old.

Over a 30-year simulation period, Prevention 2 would avert 78% of the meningococcal cases (95% prediction interval: 63%–90%) expected under the Base policy if serogroup A is not replaced by remaining serogroups after elimination, and would avert 87% (77%–93%) of meningococcal cases if complete strain replacement occurs. Compared to the Base policy and at the PMC vaccine price of US$4 per dose, strategies that use PMC vaccine (i.e., Base Prime and Preventions 1 and 2) are expected to be cost saving if strain replacement occurs, and would cost US$51 (−US$236, US$490), US$188 (−US$97, US$626), and US$246 (−US$53, US$703) per DALY averted, respectively, if strain replacement does not occur.

An important potential limitation of our study is the simplifying assumption that all circulating meningococcal serogroups can be aggregated into a single group; while this assumption is critical for model tractability, it would compromise the insights derived from our model if the effectiveness of the vaccine differs markedly between serogroups or if there are complex between-serogroup interactions that influence the frequency and magnitude of future meningitis epidemics.

**Conclusions:**

Our results suggest that a vaccination strategy that includes a catch-up nationwide immunization campaign in young adults with a PMC vaccine and the addition of this new vaccine into EPI is cost-effective and would avert a substantial portion of meningococcal cases expected under the current World Health Organization–recommended strategy of reactive vaccination. This analysis is limited to Burkina Faso and assumes that polyvalent vaccines offer equal protection against all meningococcal serogroups; further studies are needed to evaluate the robustness of this assumption and applicability for other countries in the meningitis belt.

## Abstract 2

Reza Yaesoubi and colleagues present a model that identifies health impacts and cost-effectiveness of different meningococcal vaccines and strategies in the meningitis belt in Africa.

## Author summary

**Why was this study done?:**

- Meningococcal epidemics remain a major cause of morbidity and mortality in the meningitis belt, a region in sub-Saharan Africa with an estimated population of 400 million people.
- The advent of affordable polyvalent meningococcal vaccines offers the opportunity for more effective control of *Neisseria meningitidis* in this region.
- It is not yet clear how best to use these novel polyvalent meningococcal vaccines to control meningococcal epidemics in the African meningitis belt.

**What did the researchers do and find?:**

- We developed a mathematical model that describes the key characteristics of meningococcal epidemics within districts of Burkina Faso.
- Our model estimates the health impact and costs of different meningitis vaccines and vaccination strategies (for example, in routine, reactive, or catch-up mass immunization campaigns).
- We project that a nationwide immunization campaign in young adults with a polyvalent meningococcal conjugate vaccine and the addition of this new vaccine into the Expanded Program on Immunization would be cost-effective.
- The estimated incremental cost of this new strategy is less than US$1,980 (3 times per capita gross domestic product of Burkina Faso in 2015) per disability-adjusted life year averted, compared with the current WHO-recommended strategy.

**What do these findings mean?:**

- This work suggests an opportunity to revisit the current WHO strategy for meningitis control in sub-Saharan Africa once affordable polyvalent meningococcal conjugate vaccines become available.
- Our results indicate that these novel vaccines can be used in a cost-effective manner to control meningococcal epidemics if adopted within the Expanded Program on Immunization and used in a catch-up nationwide vaccination program in Burkina Faso, but further studies are needed to evaluate the effectiveness and cost-effectiveness of this policy in other countries.

## Introduction

*N*. *meningitidis* remains a major cause of morbidity and mortality in the meningitis belt, a region in sub-Saharan Africa extending from Senegal to Ethiopia, with an estimated population of 400 million people [1]. In this region, meningitis epidemics occur sporadically, resulting in tens of thousands of cases and imposing substantial economic costs to affected communities. Since the late 1970s, control of meningitis epidemics in the meningitis belt has relied on reactive vaccination campaigns using polysaccharide vaccines. These reactive campaigns are triggered once an outbreak surpasses an epidemic threshold defined by the World Health Organization (WHO). While timely implementation of this reactive strategy may blunt the severity of meningococcal epidemics [2–4], in many settings the impact of reactive vaccination campaigns is limited by delays in the diagnosis and reporting of meningitis cases and subsequent delays in the launch of these vaccination activities [5].

In December 2010, a new group A meningococcal conjugate vaccine, PsA-TT (MenAfriVac) [6], was introduced in Burkina Faso, Mali, and Niger, and within one month, almost 20 million individuals, ages 1–29 years, were vaccinated [7–9]. The introduction of MenAfriVac across the meningitis belt has been associated with a dramatic reduction of meningitis A cases and carriage during subsequent seasons [10–12]. While MenAfriVac has been successful in controlling group A disease and transmission, persistent threat from other meningococcal serogroups [13,14] has spurred development of polyvalent vaccines that target non-A serogroups, including C, Y, W, and X [15–17].

The advent of affordable polyvalent meningococcal vaccines offers the opportunity for more effective control and potential elimination of *N*. *meningitidis* epidemics in the meningitis belt, but the health impact and costs of alternative vaccination strategies are not yet clear [18]. While available polyvalent meningococcal polysaccharide (PMP) vaccines can only be used in reactive campaigns because they are poorly immunogenic in children under 2 years old [19,20] and induce a short period of immunity in adults [1,21], polyvalent meningococcal conjugate (PMC) vaccines are immunogenic among infants and can induce longer-term protection. Therefore, PMC vaccines can potentially replace MenAfriVac in the Expanded Program on Immunization (EPI) and can also be used in reactive and/or mass preventive vaccination campaigns. Consensus on how to best use these novel polyvalent meningococcal vaccines has not yet been achieved.

Here, we describe a mathematical transmission model that captures the key characteristics of meningococcal epidemics in Burkina Faso as well as the costs associated with routine, reactive, and preventive vaccination campaigns. We utilize this model to investigate the health effects and costs associated with alternative vaccination policies that can inform the use of PMP and PMC vaccines.

## Methods

### Model structure

We developed a stochastic compartmental model of meningococcal transmission to capture the essential characteristics of meningococcal epidemics within 55 districts of Burkina Faso. This level of spatial disaggregation by district is necessary to model reactive vaccination campaigns that are triggered within each district upon passing the WHO epidemic threshold of 10 cases per 100,000 population per week [22,23]. To allow for age-specific mixing patterns and targeting of vaccinations, the simulated population was stratified into relevant age groups. We used a gravity model to describe the mixing pattern of individuals residing in different districts (see S1 Text).

**Fig 1:**
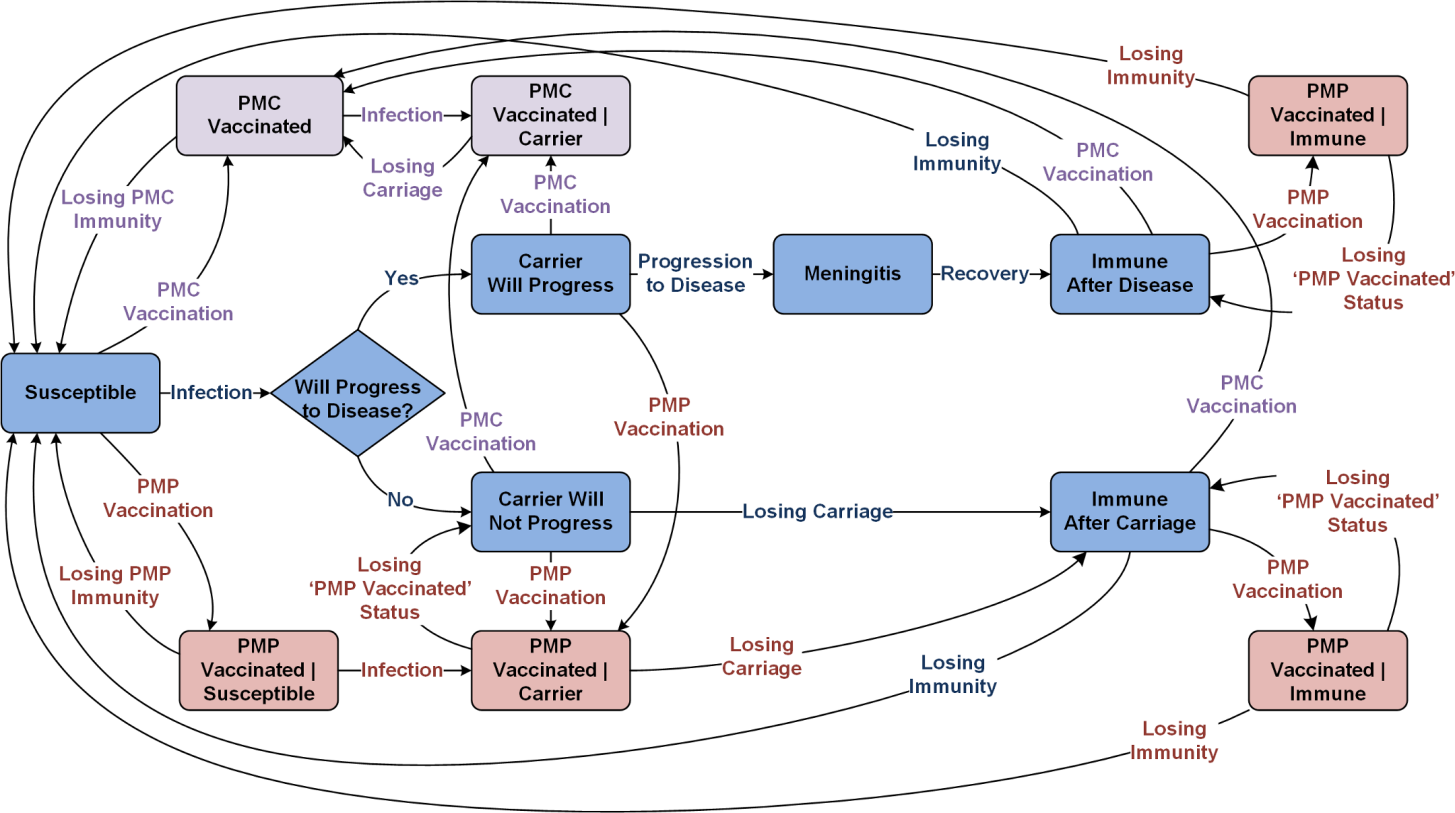
Meningitis natural history and transmission dynamics, assuming a single aggregated circulating serogroup. PMC, polyvalent meningococcal conjugate; PMP, polyvalent meningococcal polysaccharide.

The adoption of MenAfriVac as part of the EPI is expected to eliminate serogroup A meningococcal epidemics in the meningitis belt by 2020 [10,11,24–26]. Our model therefore assumes that there is no circulation of serogroup A and aggregates all remaining serogroups covered in PMC and PMP vaccines (Fig 1). Upon infection, individuals become asymptomatic carriers, but only a small portion of these infections lead to meningitis. Individuals with active disease and individuals with asymptomatic carriage contribute to the force of infection. Because the duration of carriage is relatively short [27], we assume that the probability of superinfection during carriage is negligible. Individuals who recover from active disease and carriers who clear carriage will remain temporarily immune to reinfection [27,28]. Details of our modeling approach are provided in S1 Text.

### Modeling the impact of MenAfricVac vaccine

**Fig 2:**
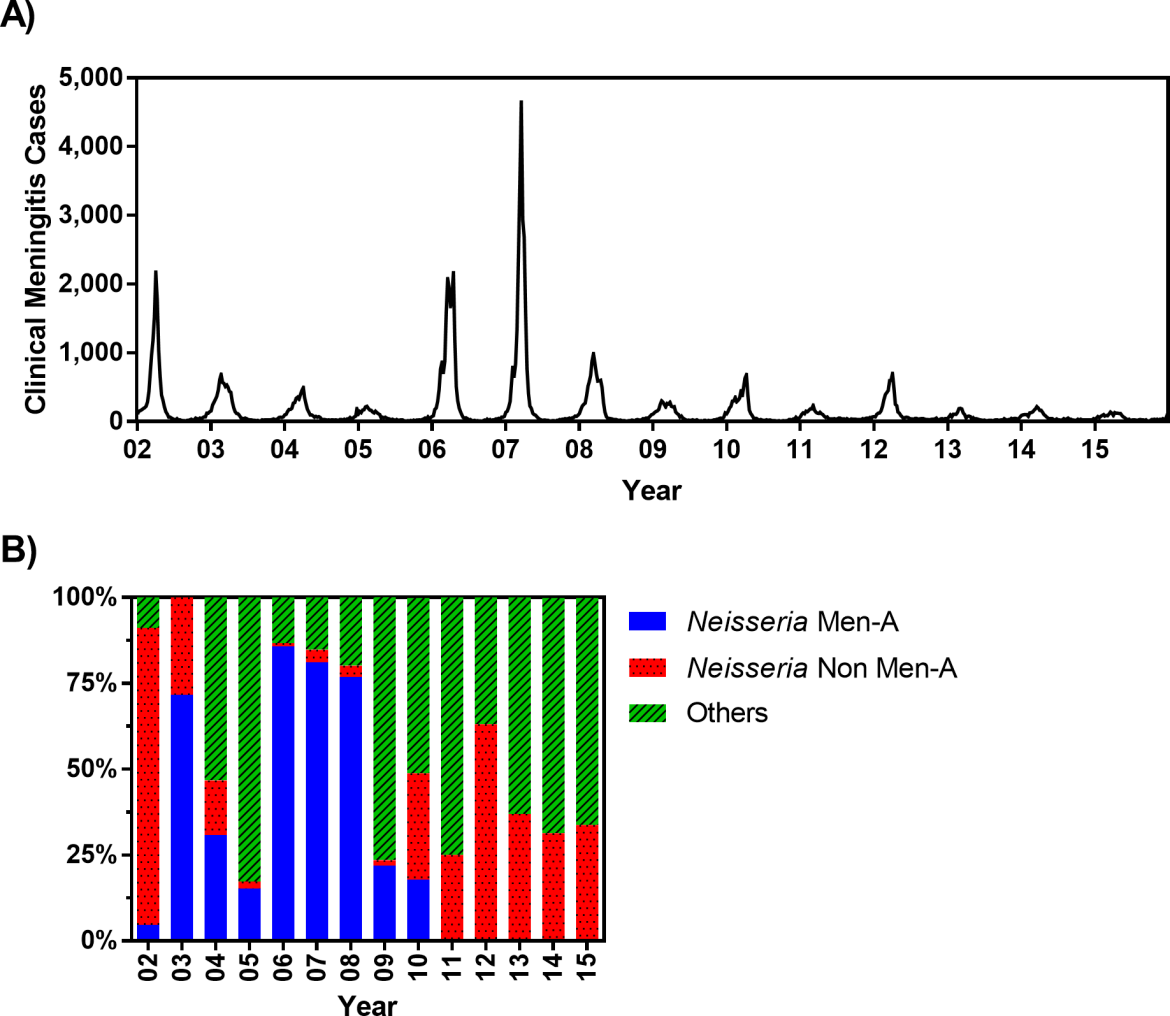
(A) Weekly clinical meningitis cases in Burkina Faso reported between 2002 and 2015 (data were made available from the Ministry of Health, Burkina Faso). (B) Percentage of confirmed meningitis cases that are associated to *Neisseria meningitis* serogroup A, *N*. *meningitidis* non-A serogroups (including C, W, and X), and other pathogens (including *Streptococcus pneumoniae* and *Haemophilus influenzae* type b) from 2002–2015. These estimates are obtained from WHO [31] (for 2002), WHO Enhanced Meningitis Bulletin (for 2003–2005), Burkina Faso *Maladies Potentiel Épidémie* (MPE) surveillance data (for 2006 and 2012–2015), and Novak et al. [11] (for 2007–2011) (see S1 Data). Men-A, meningitis serogroup A; Non Men-A, meningitis serogroups other than A.

Because the immunity induced by MenAfriVac is serogroup specific, the possibility of strain replacement due to the rollout of MenAfriVac across the meningitis belt cannot yet be excluded [29,30]. Therefore, to account for the impact of MenAfriVac on future epidemics, we consider two extreme scenarios: (1) “with strain replacement” reflects the pessimistic assumption of essentially complete strain replacement and assumes that future epidemics will occur with similar frequency and magnitude after the introduction of MenAfriVac (Fig 2A) and (2) “without strain replacement” assumes that future serotype incidence will be similar to the past but with serogroup A excluded (i.e., potentially lower frequency and/or magnitude of epidemics). For the latter scenario (without strain replacement), we determined the weekly meningitis incidence using estimates for the proportion of confirmed meningitis cases during 2002–2015 due to non-serogroup A *N*. *meningitidis* (Fig 2B). Evaluation of vaccine strategies under these two extreme scenarios allows us to identify crude bounds on the performance of vaccine policies, given existing uncertainty about the degree to which strain replacement can be expected.

### Data sources and model calibration

**Fig 3:**
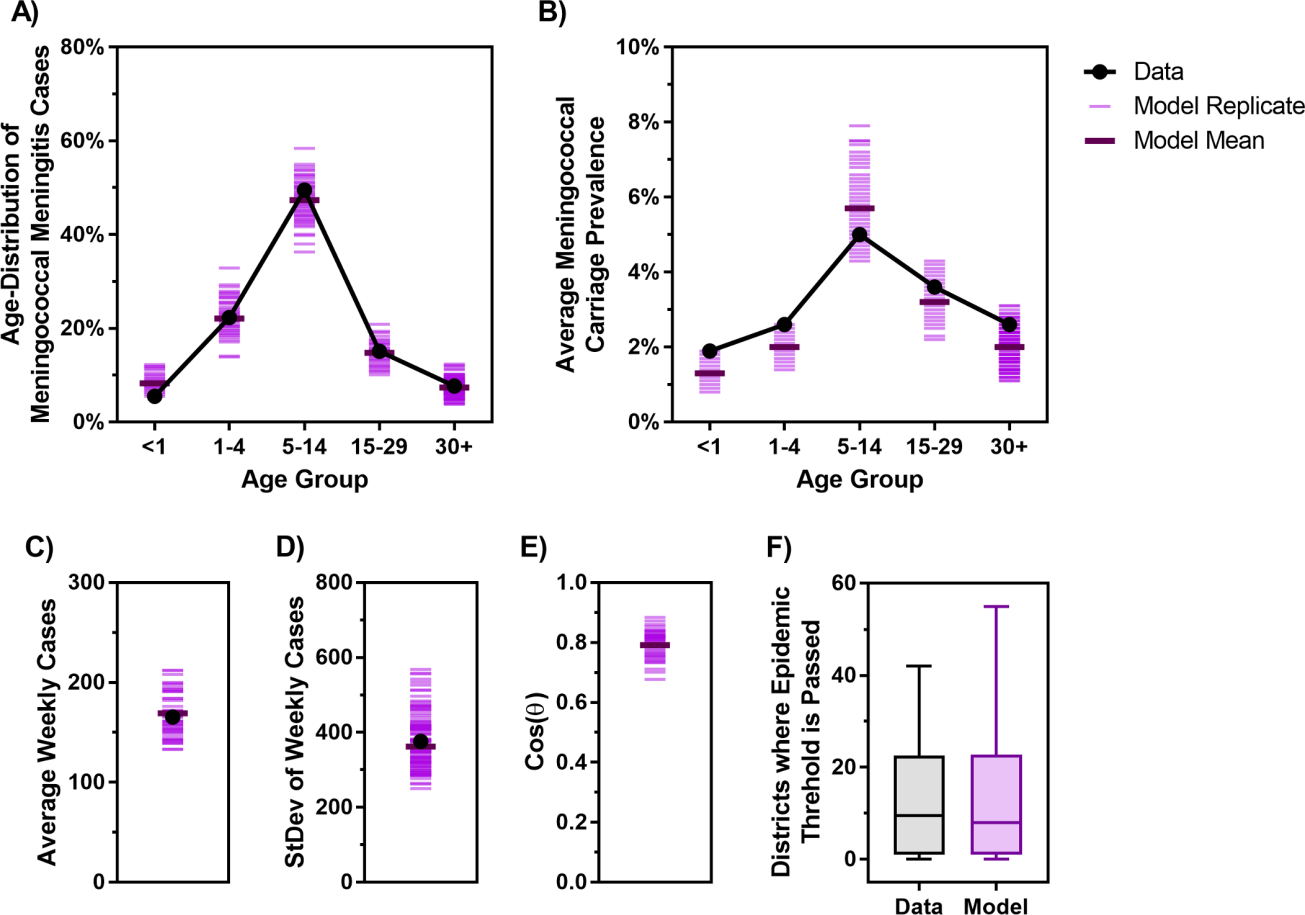
The proposed model matches the key characteristics of meningitis epidemics in Burkina Faso observed between 2002 and 2015. (A) Age distribution of probable meningococcal meningitis in Burkina Faso from 2007–2011 [11] versus the age distribution of cases generated by the model. (B) Estimated meningococcal carriage prevalence in different age groups from carriage survey studies in the African meningitis belt [34] versus the age-specific average carriage prevalence obtained from the model. (C–D) Average and standard deviation of weekly clinical meningitis cases observed from 2002–2015 versus those produced by the model. (E) Cosine of the angle (***θ***) between the vectors of Fourier amplitude for observed and simulated meningitis time series (cosine of 1 indicates total match in periodicity and cosine of 0 indicates no overlap between the significant periods of two time series; see S1 Text for additional details). (F) Observed (Data) and simulated (Model) number of districts in each year between 2002 and 2015 in which the threshold of 10 meningitis cases per 100,000 population was exceeded. Cos, cosine; StDev, standard deviation.

The age-specific mortality rate and life expectancy are informed by population data (S1 Text) [32,33]. The weekly clinical meningitis notification data are provided by Burkina Faso’s Ministry of Health (Fig 2A). Our model is calibrated against the age distribution of meningococcal meningitis incidence (Fig 3A) and average age-specific meningococcal carriage prevalence (Fig 3B). Because reactive campaigns are launched when the number of clinical meningitis cases exceeds the WHO epidemic threshold [22,23], the model must reproduce observed trends in clinical diagnoses (Fig 2A). To this end, we used the average, standard deviation, and periodicity (as characterized by Fourier analysis) of the time series of clinical diagnoses as additional calibration targets (Fig 3C–3E). Finally, Fig 3F demonstrates that the number of districts in which the epidemic threshold of 10 meningitis cases per 100,000 persons has been exceeded in our simulations is consistent with the epidemics observed between 2002 and 2015 in Burkina Faso.

**Fig 4:**
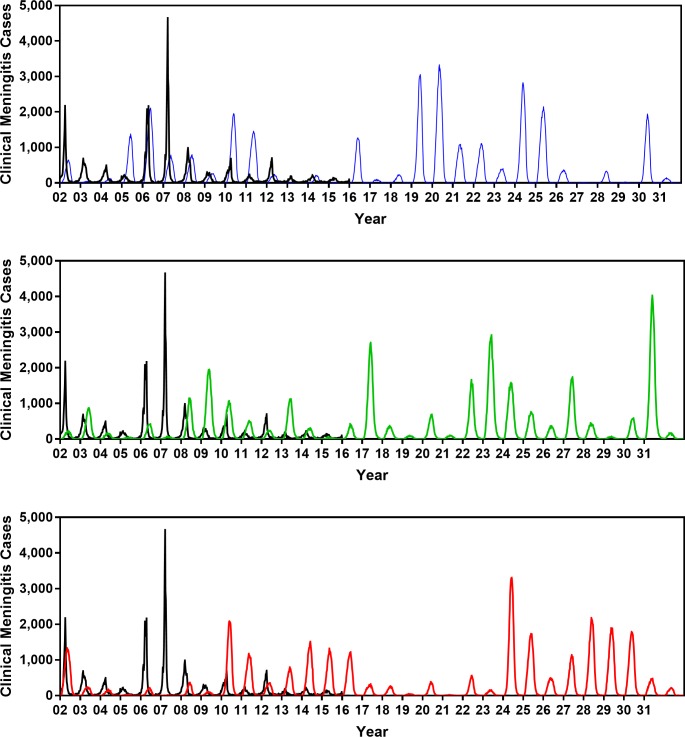
Comparing the clinical meningitis time series observed between 2002 and 2015 in Burkina Faso (black curve) with three simulated trajectories produced by the calibrated model (blue, green, and red curves). The periodicity at which simulated epidemics are occurring matches the periodicity of observed epidemics. Fig 3 shows that trajectories generated by our model also match other key properties of meningococcal epidemics in Burkina Faso (e.g., age distribution of cases, age-specific carriage prevalence, average weekly incidence, and number of districts in each year between 2002 and 2015 in which the threshold of 10 meningitis cases per 100,000 population was exceeded).

Fig 4 displays the clinical meningitis time series from three simulated trajectories over 30 years produced by the calibrated model, in comparison with the clinical meningitis time series observed in Burkina Faso during 2002–2015. We emphasize that our goal is not to fit to the timing of past epidemics but instead to calibrate the model against the periodicity of past epidemics, in addition to calibration targets depicted in Fig 3. Details of our calibration approach are described in S1 Text.

### Alternative vaccine policies

Even after the introduction of MenAfriVac in 2010, reactive strategies using PMP vaccine, alongside the EPI with MenAfriVac, remain important for responding to epidemics caused by non-A meningococci (Base strategy in Table 1) [35]. Because PMP vaccine is poorly immunogenic in infants and children younger than 2 years old [15], an alternative strategy is to use PMC vaccine in place of both MenAfriVac in EPI and PMP vaccines in reactive campaigns (Base Prime strategy in Table 1). Expanding PMC vaccine coverage to older age groups through preventive vaccination campaigns can both mitigate the risk of meningococcal epidemics by reducing the size of the at-risk population and can potentially lead to the elimination of meningitis (Prevention 1 and 2 strategies in Table 1). While the Base Prime strategy attempts to contain local outbreaks by implementing district-level reactive campaigns, the two Prevention strategies seek to reduce the population risk of infection through preventive campaigns implemented at a national level. We note that Base Prime can potentially lead to the same level of herd immunity as Prevention strategies over the long term, but this occurs only after the population has aged sufficiently such that all young adults were vaccinated through the EPI program or after outbreaks have occurred in a sufficient number of districts.

**Table 1:** Alternative vaccine strategies for employing polyvalent meningococcal vaccines (post 2020).

Strategy	Routine Vaccination (EPI)	Reactive Campaign	Preventive Campaign
Base	MenAfriVac at 9 mo	PMP vaccine for 1–29-yo	-
Base Prime	PMC vaccine at 9 mo	PMC vaccine for 1–29-yo	-
Prevention 1^#^	PMC vaccine at 9 mo	-	PMC vaccine for 1–18-yo
Prevention 2^#^	PMC vaccine at 9 mo	-	PMC vaccine for 1–29-yo

^#^For Prevention 1 and 2 strategies, we assume that reactive campaigns using PMC vaccine would still occur if the epidemic threshold is exceeded.

EPI, Expanded Program on Immunization; PMC, polyvalent meningococcal conjugate (novel vaccine); PMP, polyvalent meningococcal polysaccharide; yo, year-old.

Our model assumes that vaccinated individuals are protected against progression to invasive disease for a limited time (Fig 1), but the level and duration of protection differ for PMP and PMC vaccines. We assume that PMP- and PMC-vaccinated carriers still remain infectious and contribute to the force of infection. Because the duration of immunity provided by PMP vaccination is rather short (Table 2), we assume that PMP vaccination does not impact carriage- or disease-induced immunity. Upon vaccination with PMP, individuals with carriage- or disease-induced immunity move to PMP-Vaccinated compartments to prevent revaccination but will either return to the corresponding Immune compartments at the beginning of the following epidemic season or lose their carriage- or disease-induced immunity and join the Susceptible compartment.

**Table 2:** Vaccination assumptions.

Parameter	Value	Data Sources
Relative susceptibility to infection (compared with unvaccinated)		
PMP vaccine	0%–15%	[1,17,19,36,37]
PMC vaccine	0%–10%	[38,39]
Duration of protection		
PMP vaccine		
Age 1–4 years	1–3 years	[19,20]
Age 5+ years	3–5 years	[1,21]
PMC vaccine	10–20 years	[28,39]
Delay between administration of PMP or PMC and the establishment of immunity	2 weeks	[40]
Vaccine uptake		
Routine vaccination^#^	80%–90%	[41]
Preventative and reactive vaccination (within 10 days)	90%–100%	[7–9]

^#^Vaccine uptake for routine vaccination was assumed to be equal to the 9-mo measles coverage [41].

PMC, polyvalent meningococcal conjugate; PMP, polyvalent meningococcal polysaccharide.

For the Base and Base Prime strategies, once an epidemic is declared in a district, a vaccine procurement order will be placed. For each district, we assume that the delay between exceeding the epidemic threshold and the initiation of a reactive vaccination campaign follows a discrete Uniform distribution [2, 10] weeks (Fig F in S1 Text). For Prevention strategies, a catch-up vaccination campaign is launched in November of the first simulation year and is completed before the start of the next epidemic season. During this period, PMC vaccine will be available to all individuals who are eligible for this catch-up vaccination.

### Health and financial outcomes of vaccination strategies

The costs of vaccination programs in Burkina Faso are borne by the government and donors; hence, demonstration of affordability is essential for these programs to be considered in practice. We therefore take the payer’s perspective in estimating the costs associated with vaccine strategies. Our model accounts for costs incurred because of meningitis case management; care for patients who experience sequelae; and the operation costs of routine, reactive, and preventive vaccination campaigns. We assumed US$0.49 per MenAfriVac dose and US$4 per PMP vaccine dose [42]; as the price of the PMC vaccine is not yet determined, we allow this price to vary from US$4 to US$10 in sensitivity analyses. To measure the health outcome associated with each vaccine strategy, we use disability-adjusted life years (DALYs) [43]. Both costs (presented in the US dollars) and health outcomes are discounted at an annual rate of 3% to 2016. The details of the cost and DALY calculations are provided in S1 Text.

We followed the Consolidated Health Economic Evaluation Reporting Standards (CHEERS) [44] to report the results of our cost-effectiveness analysis study (see S11 in S1 Text). All estimates from the model are presented as the average and 95% prediction intervals (the 2.5th and 97.5th percentiles) of 500 epidemic trajectories simulated over a 30-year period. The number of simulated trajectories was chosen such that the resulting prediction intervals were stable (i.e., the values of the 2.5th and 97.5th percentiles of estimates did not vary appreciably when additional trajectories were used). Costs and health effects of vaccinations strategies are presented with respect to the current WHO-recommended strategy of reactive vaccination campaigns using PMP vaccines (Base strategy in Table 1).

## Results

Over a 30-year simulation period in Burkina Faso using the Base strategy of the EPI with MenAfriVac and reactive immunization with PMP vaccines, we project an annual average of 5,412 meningococcal cases (95% prediction interval: 105–16,550) with strain replacement and 1,642 (32–5,794) meningococcal cases without strain replacement. Compared to a counterfactual scenario in which reactive vaccination is not used, this represents an expected reduction of 45% (26%–62%) and 43% (22%–59%) in meningococcal incidence. The relatively modest impact of this strategy is attributable to (1) delays in the launch of reactive campaigns within districts upon crossing the epidemic threshold and (2) the short duration of immunity and lack of effect on carriage offered by PMP vaccines.

**Fig 5:**
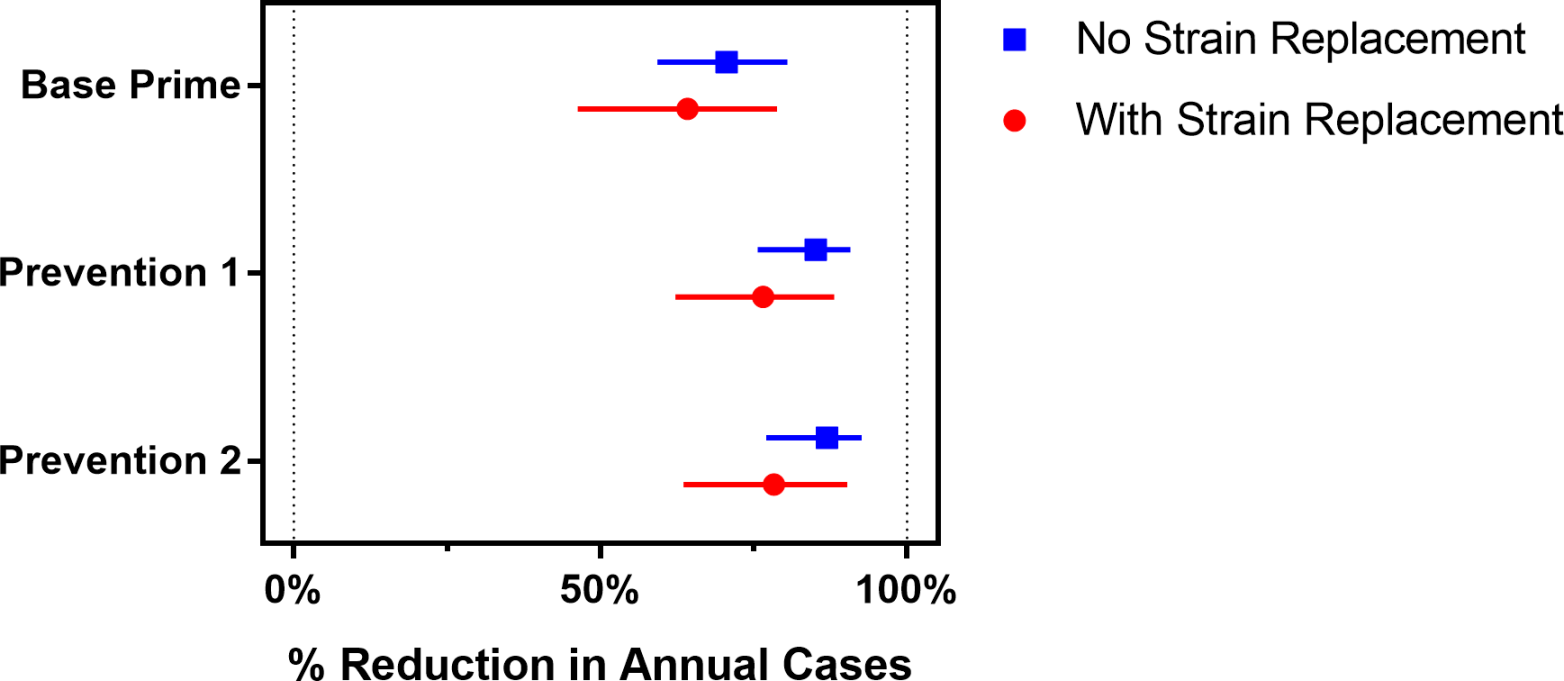
The expected percentage reduction in annual meningococcal cases over a 30-year simulation period for the vaccination strategies described in Table 1, compared to the Base strategy. Bars represent the 95% prediction intervals.

**Fig 6:**
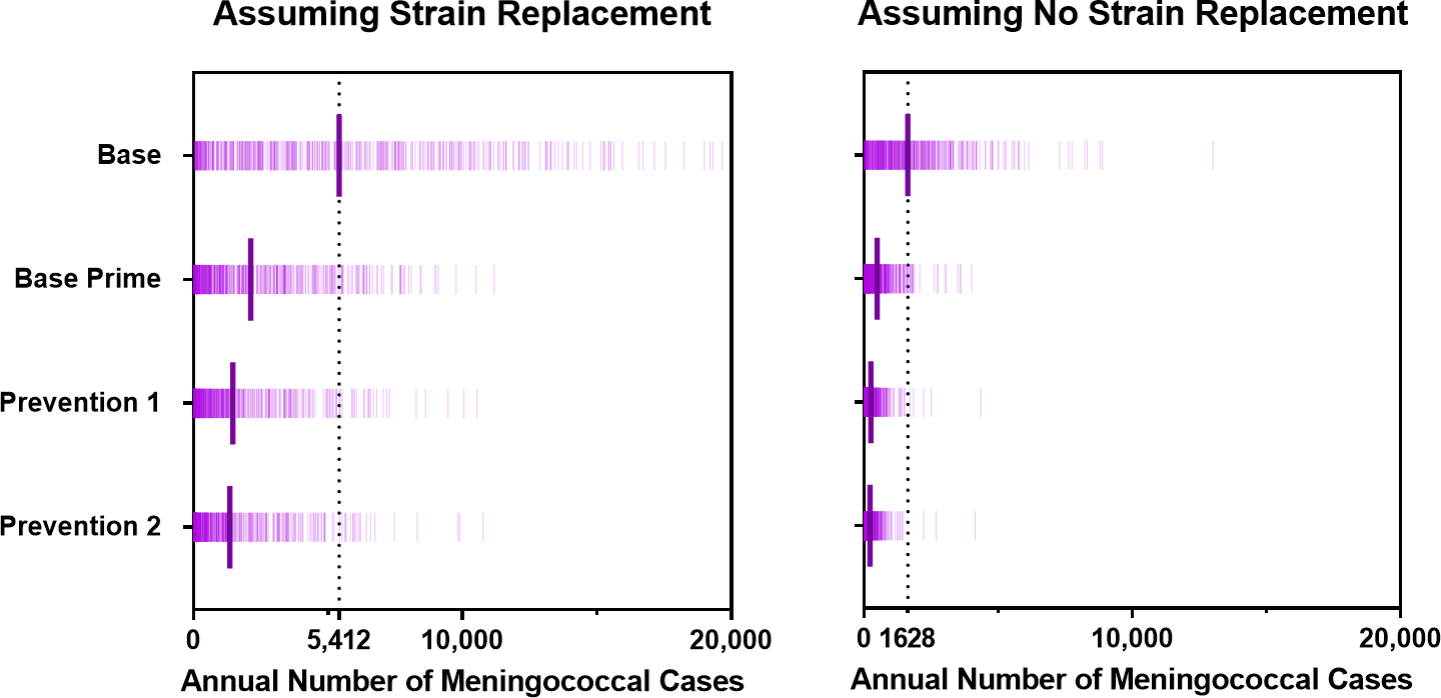
Annual number of meningococcal cases for scenarios with and without strain replacement projected by the model under the vaccination strategies described in Table 1. The long bars represent the average annual number of meningococcal cases expected under each strategy option.

Vaccination strategies that use PMC vaccines could markedly reduce the expected annual number of meningitis cases (Fig 5), but they do not eliminate the possibility of meningitis outbreaks (Fig 6). The Prevention 2 strategy results in the most dramatic impact, averting 78% (63%–90%) of cases expected to occur under the Base strategy if strain replacement occurs and averting 87% (77%–93%) if no strain replacement occurs. Our model suggests that under strategies that utilize PMC vaccines, meningitis outbreaks may recur 10–15 years after the implementation of the first mass preventive campaign, when the immunity induced by PMC vaccines begins to wane in the adult population (Fig G in S1 Text).

**Fig 7:**
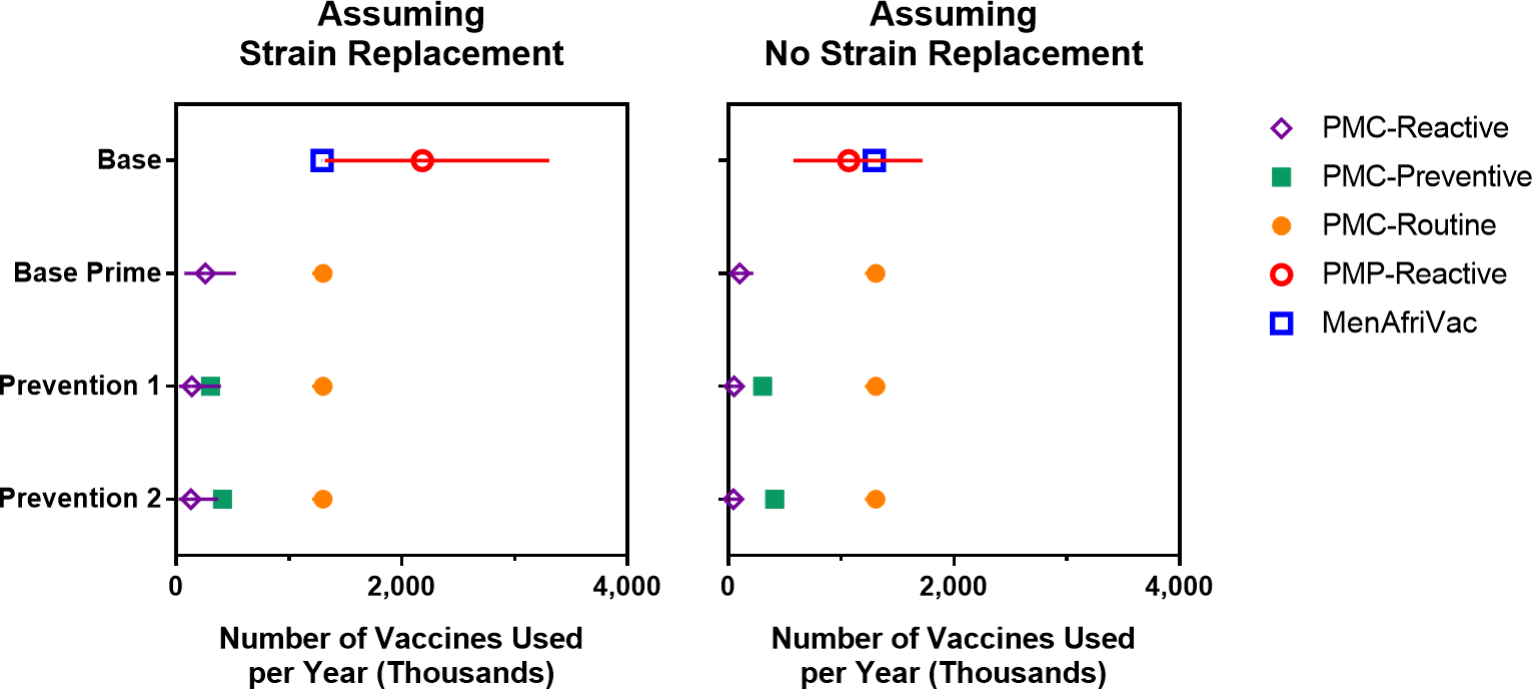
Expected number of vaccines used per year (over a 30-year simulation period) for scenarios with and without strain replacement. Error bars represents 95% projection intervals (error bars that are shorter than the width of symbols are not shown). PMC, polyvalent meningococcal conjugate; PMP, polyvalent meningococcal polysaccharide.

Fig 7 displays the expected number of vaccines required for each vaccination strategy. Under either strain-replacement assumption, the Base strategy has the highest expected annual consumption of total vaccine doses and the Base Prime strategy has the lowest expected annual consumption of vaccine doses. The wide prediction intervals for the estimated number of PMP vaccines used under the Base strategy are the result of the sporadic occurrence of meningococcal outbreaks that may trigger district-wide reactive campaigns. We also note that extending the projection horizon does not impact the estimated annual consumption of MenAfriVac, PMP, or PMC vaccines in routine programs, but it reduces the estimated annual consumption of PMC vaccines in reactive and mass preventive campaigns. This is because preventive campaigns are implemented a single time at the beginning of the projection period, and the outbreaks under the Base Prime strategy occur only in early years, when there is a pool of children and young adults who were born too early to receive PMC vaccine in their routine infant vaccination schedules.

**Fig 8:**
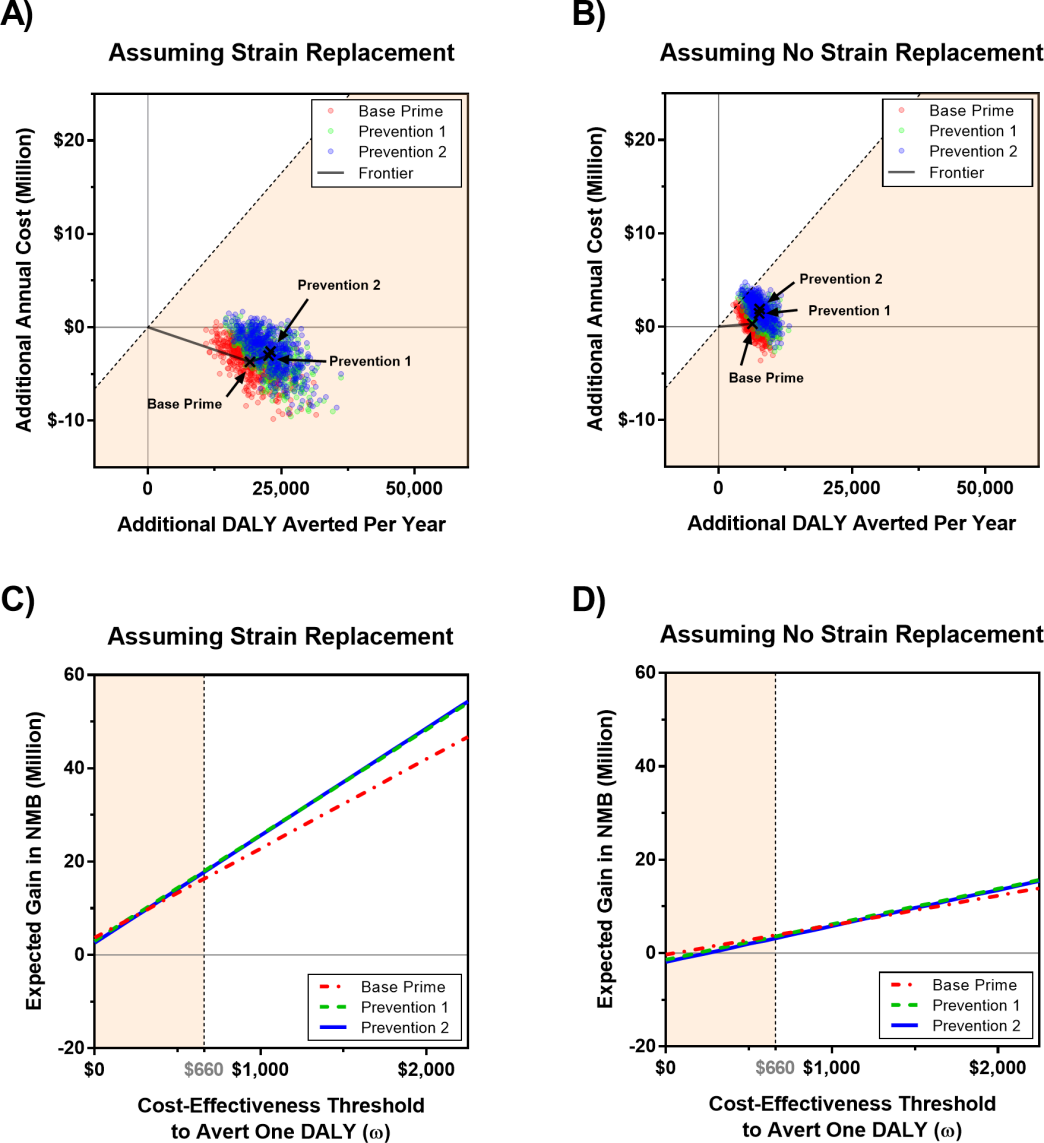
Economic evaluation of vaccine strategies described in Table 1 for scenarios with and without strain replacement in which the price of PMP and PMC vaccines are US$4 per dose (see S1 Text for sensitivity analysis to the vaccine prices). In cost-effectiveness panels (A) and (B), each dot represents the additional cost and DALYs averted in a simulated trajectory with respect to the Base strategy, which represents the current WHO policy that relies on reactive vaccination campaigns using PMP vaccines in districts in which the epidemic threshold is passed. The x’s represent the expected additional cost and DALYs averted with respect to the Base strategy. Panels (C) and (D) show the expected gain in NMB of a strategy with respect to the Base strategy for a given cost-effectiveness threshold, ω. The diagonal dashed line in panels (A) and (B) and the vertical dashed line in panels (C) and (D) represents the cost-effectiveness threshold of one per capita gross domestic product of Burkina Faso, which is estimated to be US$660 in 2015 [48]. All costs and DALYs are discounted at rate 3% to year 2016. DALY, disability-adjusted life year; NMB, net monetary benefit; PMC, polyvalent meningococcal conjugate; PMP, polyvalent meningococcal polysaccharide.

For the scenario with strain replacement, the Base strategy is dominated by Base Prime, as the latter strategy is expected to cost less and reduce the population’s DALYs (Fig 8A and Table 3). If strain replacement does not occur, we estimate the incremental cost-effectiveness ratios (ICERs) for DALYs averted by Base Prime compared with Base at US$51 (−US$233–US$476). Per WHO recommendations, strategies that avert one DALY for less than the per capita gross domestic product (GDP) are considered “very cost-effective” and one DALY for less than three times the per capita GDP as “cost-effective” [45]. Hence, at the cost-effectiveness threshold of US$660, the per capita GDP of Burkina Faso in 2015, the Base Prime strategy is considered cost-effective with respect to the Base strategy under either strain-replacement scenario.

**Table 3:** Cost-effectiveness of vaccination strategies, assuming a vaccine cost of US$4 per dose for PMP and PMC vaccines.

Strategy	Expected Annual Cost in Million US$	Expected Annual DALYs	Expected Incremental Annual Cost in Million US$	Expected Incremental Annual DALYs	ICER in US$ per DALY Averted
	Assuming Strain Replacement				
Base	10.2 (6.7–15.1)	28,956 (19,260–39,108)	−	−	Dominated
Base Prime	6.5 (5.5–7.9)	9,809 (4,820–17,482)	−3.7 (−7.9–−0.6)	19,147 (12,896–26,764)	Cost Saving
Prevention 1	7.2 (6.5–8.2)	6,306 (2,291–12,590)	−3.0 (−7.5–0.2)	22,650 (16,472–29,966)	188 (−6–402)
Prevention 2	7.6 (7.0–8.6)	5,963 (2,044–12,128)	−2.6 (−7.1–0.6)	22,993 (16,597–30,301)	1,291 (−8,058–7,359)
	Assuming No Strain Replacement				
Base	5.4 (3.4–7.9)	8,773 (5,535–12,871)	−	−	−
Base Prime	5.7 (5.2–6.4)	2,436 (1,266–4,498)	0.3 (−2.0–2.2)	6,337 (3,877–9,079)	51 (−233–476)
Prevention 1	6.8 (6.4–7.2)	1,170 (548–2,685)	1.4 (−1.0–3.4)	7,603 (4,879–11,013)	870 (483–1,599)
Prevention 2	7.2 (6.8–7.7)	1,062 (467–2,595)	1.9 (−0.5–3.9)	7,711 (4,990–11,115)	4,356 (−40,482–44,697)

Numbers in parentheses are 95% prediction intervals.

Expected incremental annual cost, DALYs, and ICER for each strategy are calculated with respect to the left-hand side strategy on the cost-effectiveness frontier shown in Fig 8 (i.e., Base Prime compared to Base; Prevention 1 compared to Base Prime; and Prevention 2 compared to Prevention 1).

DALY, disability-adjusted life year; ICER, incremental cost-effectiveness ratio; PMC, polyvalent meningococcal conjugate; PMP, polyvalent meningococcal polysaccharide.

The ICER of Prevention 1 compared to Base Prime is estimated at US$188 (**−**US$6–US$402) and US$870 (US$483–US$1,599) per DALY averted for with and without strain replacement, respectively. These ICER estimates are below the cost-effectiveness threshold of US$1,980, three times the per capita GDP of Burkina Faso in 2015. Compared with the Base policy, strategies that use PMC vaccine (i.e., Base Prime and Preventions 1 and 2) are expected to be cost saving if strain replacement occurs and would cost US$51 (**−**US$236, US$490), US$188 (**−**US$97–US$626), and US$246 (**−**US$53–US$703) per DALY averted if strain replacement does not occur.

We also compare the performance of vaccination strategies in terms of their impact on the population’s net monetary benefit (NMB) [46,47] for varying values of cost-effectiveness threshold (ω). The expected gain in NMB of a strategy is calculated with respect to the Base strategy as ω × (additional DALYs averted by the strategy)–(additional cost of the strategy). Fig 8C and 8D confirms that strategies that use the PMC vaccine dominate Base and that Prevention 1 and 2 strategies demonstrate similar performance under both strain-replacement scenarios. As expected, the cost-effectiveness of the vaccine strategies varies between strain-replacement scenarios and all strategies—Base Prime, Prevention 1, and Prevention 2—present larger incremental benefit when strain A elimination is followed by complete strain replacement (Fig 8 and Table 3).

Our sensitivity analysis shows that reducing PMP vaccine price from US$4 to US$2 per dose does not change the conclusions about the comparative performance of these vaccination strategies (Fig H in S1 Text). While increasing the price of PMC vaccine from US$4 to US$10 per dose diminishes the cost-effectiveness of vaccination strategies that involve PMC vaccines, these strategies maintain their relative performance with respect to the Base policy (Fig I in S1 Text). If PMC vaccine price is US$10 per dose, we estimate an average cost per DALY averted by Base Prime and Prevention 1 and 2 strategies with respect to the Base strategy at US$257 (US$57–US$558), US$286 (US$84–US$546), and US$326 (US$117–US$602) when strain replacement occurs and at US$1,246 (US$631–US$2,336), US$1,369 (US$759–US$2,431), and US$1,488 (US$833–US$2,619) without strain replacement.

## Discussion

While the currently recommended strategy for meningitis control in sub-Saharan Africa relies on reactive vaccination campaigns using PMP vaccines in districts where the epidemic threshold is passed, our model suggests that this approach will be outperformed by alternative policies using affordable PMC vaccines. The use of PMC vaccines in the EPI and in reactive vaccination programs could markedly reduce the public health burden of meningococcal epidemics but still leaves districts at substantial risk of sporadic outbreaks. The addition of nationwide catch-up vaccination campaigns to immunize 1–18-year-olds with PMC vaccines could prevent the majority of meningococcal cases. Our results suggest that this strategy is likely to be cost-effective (and potentially cost saving) with respect to the current WHO-recommended meningitis control strategy in sub-Saharan Africa once affordable PMC vaccine becomes available.

The introduction of MenAfriVac is expected to eliminate serogroup A meningitis in the meningitis belt [10,11,24–26], but little is known about the impact of MenAfriVac on the future non-A epidemics. As expected, benefits of additional vaccination interventions are highest when the elimination of serogroup A is followed by replacement by other circulating serogroups. While we do not know the likelihood or extent of serogroup replacement, our analysis shows that the comparative performance of the vaccination strategies we considered are not meaningfully altered by this source of uncertainty. Nevertheless, sustaining strong case-based surveillance in the post-MenAfriVac will facilitate more accurate estimates of the health impact and costs of these competing strategies.

Meningococcal outbreaks in the meningitis belt are sporadic and caused by different serogroups (mainly A, C, W, and X) [11,49], and the accurate prediction of future meningitis epidemics is challenged by the absence of data to characterize competition between these serogroups [50]. Most meningitis transmission models either describe the circulation of a single serogroup or two serogroups (e.g., vaccine type and non-vaccine type) [27,28,50–53]. In our study, we assume that polyvalent vaccines will offer protection against all meningococcal serogroups that can circulate in this setting, and therefore our model aggregates all serogroups into a single vaccine type serogroup. This simplification improves tractability but would compromise the insights derived from this model if there is differential effectiveness of the vaccine by serogroup or if there are complex between-serogroup interactions that influence the frequency and magnitude of future meningitis epidemics.

While our analysis is limited to Burkina Faso, the conclusions may well apply to other hyperendemic areas in the meningitis belt, including Mali, Niger, Chad, and Northern Nigeria, as the key characteristics of meningococcal epidemics in these regions (e.g., frequency of epidemics, age distribution of cases, and age-specific carriage prevalence) are similar to those in Burkina Faso [1,54]. However, additional studies are needed to confirm the generalizability of our conclusions to other settings.

This work suggests that there is a need to revisit the current WHO strategy for meningitis control in sub-Saharan Africa once affordable PMC vaccines become available. Our model-based results indicate that PMC vaccines can be used in a cost-effective manner to control meningococcal epidemics if adopted within the EPI and used in a catch-up preventive vaccination program.

## Supporting information

S1 Text Additional model details and results of sensitivity analyses. (PDF)

S1 Data Data to inform model parameters and calibration targets. (XLSX)

## Abbreviations

CHEERS: Consolidated Health Economic Evaluation Reporting Standards
DALY: disability-adjusted life year
EPI: Expanded Program on Immunization
GDP: gross domestic product
ICER: incremental cost-effectiveness ratio
PMC: polyvalent meningococcal conjugate
PMP: polyvalent meningococcal polysaccharide
WHO: World Health Organization
