# Supplementary material for: The cost-effectiveness of alternative vaccination strategies for polyvalent meningococcal vaccines in Burkina Faso: A transmission dynamic modeling study
Source: PLoS Med. 2018 Jan 24;15(1):e1002495. doi: 10.1371/journal.pmed.1002495 (PMC5783340; doi:10.1371/journal.pmed.1002495)
Supplement: S1 Text — (PDF) [file pmed.1002495.s001.pdf]

S1 Text: Additional Model Details and Results of Sensitivity Analyses

S1. Burkina Faso Population Structure

The estimated population of Burkina Faso is 18,931,686 with a growth rate of 3.03%, and 42.03 births/1,000 population in 2015 [1]. We use Burkina Faso's population life tables [2] and the CIA fact book [1] to estimate the proportion of the population in age groups {<1, 1-4, 5-14, 15-18, 19-29, 30+ years old}, and age-stratified mortality rate and life expectancy (Fig A). Our model describes meningitis epidemics within 55 districts in Burkina Faso. The 2007 population and the coordinates of these districts are provided in the companion Excel file (*S2_Data*). The populations in each district age group are calculated by multiplying the district total population by the age distribution estimates shown in Fig A(A).

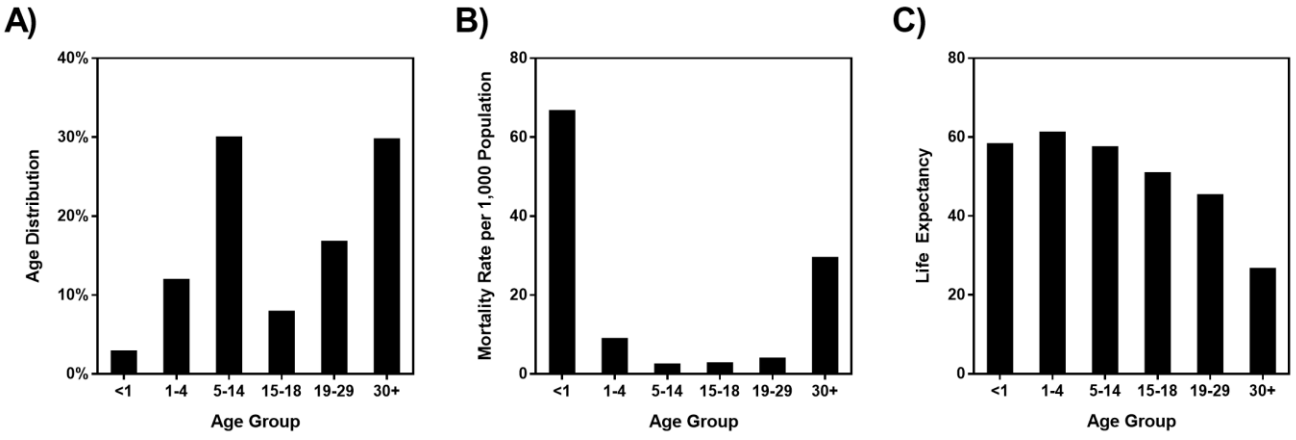

Fig A: Burkina Faso population characteristics

S2. Transmission Model

Simulation Framework

To construct the model, we introduce the following notation:

- $i \in \{1, 2, 3, 4, 5, 6\}$ index of age groups {<1, 1-4, 5-14, 15-18, 19-29, 30+};
- $k \in \{1,2, \ldots ,55\}$ index of Burkina Faso districts;
- t: epidemic time;
- $N_{(k,i)}(t)$: number of individuals in age group i of district k;
- $S_{(k,i)}(t)$: number of susceptibles in age group i of district k;

– $C_{(k,i)}(t)$: number of carriers in age group i of district k who will not develop meningitis;

– $C'_{(k,i)}(t)$: number of susceptibles in age group i of district k who will develop meningitis;

– $M_{(k,i)}(t)$: number of meningitis cases in age group i of district k;

– $I_{(k,i)}(t)$: number of members in age group i of district k who retain immunity after clearing the carriage state;

– $I'_{(k,i)}(t)$: number of susceptibles in age group i of district k who gained immunity after recovering from meningitis disease;

The state of the meningococcal epidemic at any given time t can be identified by a discrete-time Markov chain $\{s_t = (S_{(k,i)}(t), C_{(k,i)}(t), C'_{(k,i)}(t), M_{(k,i)}(t), I_{(k,i)}(t), I'_{(k,i)}(t), i \in \{1,2,3,4,5,6\}, k \in \{1,2, \ldots, 55\}\}$. Those who die from the disease leave the population and no longer contribute to disease transmission. Hence, the epidemic state s_t does not include the total number of deaths.

To generate epidemic trajectories for this model, we use Monte Carlo simulation to sample from the Markov chain $\{s_t: t = 0, \Delta t, 2\Delta t, 3\Delta t, \ldots\}$ using the following approach. Consider a particular compartment Z in which members depart due to J events. For example, members of Susceptible compartment may leave due to 1) natural death, 2) infection, 3) PMC vaccination, or 4) PCP vaccination (i.e. $J = 4$) (see Fig 1). If the number of individuals in compartment Z at time t is $Z(t)$, then the number of individuals that leave this compartment due to events $j \in \{1,2, \ldots, J\}$ follows a multinomial distribution with total counts of $Z(t)$ and probabilities $(p_0, p_1, p_2, \ldots p_J)$, where $p_0 = 1 - e^{\sum_{j=1}^{J} \mu_j \Delta t}$ is the probability of not leaving the compartment Z during $[t, t + \Delta t]$, and $p_j = \frac{\mu_j}{\sum_{j=1}^{J} \mu_j \Delta t} e^{\sum_{j=1}^{J} \mu_j \Delta t}$ is the probability of leaving the compartment Z during $[t, t + \Delta t]$ due to event $j \in \{1,2, \ldots, J\}$. We assume that the number of births in district k during $[t, t + \Delta t]$ follows a Poisson distribution with rate equal to the Burkina Faso birth rate (i.e. 42.03 births/1,000 [1]) times the population of district k at time t.

When evaluating vaccination strategies, the Markov chain $\{s_t\}$ can be augmented by additional compartments needed to capture the effect of vaccination (see Fig 1). The augmented Markov chain can still be evaluated using the method described above. To identify the new epidemic state at the next time step (i.e. $s_{t+\Delta t}$), we first sample from the multinomial distributions associated to each compartment and determine the number of births in each district, and then use these realizations to calculate the new epidemic state given the current epidemic state s_t. The events that drive the epidemic are displayed in Fig 1. For example, the number of susceptibles in age group i of district k at time $t + \Delta t$ can be calculated as:

$$
\begin{aligned}
&S_{(k,i)}(t + \Delta t) = S_{(k,i)}(t) \\
&- natrual\ deaths\ in\ age\ group\ i\ of\ district\ j \\
&- number\ aged\ to\ age\ group\ i + 1\ of\ district\ j
\end{aligned}
$$

$$- new\ infections\ in\ age\ group\ i\ of\ district\ j$$
$$- PMC\ vaccianted\ in\ age\ group\ i\ of\ district\ j$$
$$- PMP\ vaccinated\ in\ age\ group\ i\ of\ district\ j$$
$$+ births\ in\ district\ j\ (for\ i = 1)$$
$$+ number\ aged\ from\ age\ group\ i-1\ of\ district\ j$$
$$+ lost\ immunity\ after\ disease\ in\ age\ group\ i\ of\ district\ j$$
$$+ lost\ immunity\ after\ carriage\ in\ age\ group\ i\ of\ district\ j$$
$$+ lost\ immunity\ from\ PMC\ vaccine\ in\ age\ group\ i\ of\ district\ j$$
$$+ lost\ immunity\ from\ PMP\ vaccine\ in\ age\ group\ i\ of\ district\ j.$$

A thorough study of behavior and properties of models with similar sets of compartments to describe the meningitis epidemics in South Africa is presented by Irving et al. [3].

Calculating the Rate of Infection

We calculate the daily rate of infection at time t for susceptible members in age group i of district k as:

$$\mathcal{F}_{k,i}(t) = \beta_{k,i}(t) \sum_{(k',i')} \lambda_{(k,i)\leftarrow(k',i')} \frac{C_{(k',i')}(t) + C'_{(k',i')}(t)}{N_{(k',i')}(t)}, \tag{1}$$

where $\beta_{k,i}(t)$ is the transmission parameter for age group i in district k, and $\lambda_{(k,j)\leftarrow(k',j')}$ is the daily rate at which an average individual in age group i of district k contact with individuals in age group i' of district k' (see below for how $\lambda_{(k,j)\leftarrow(k',j')}$ is estimated). We let $\beta_{k,i}(t) = \gamma_k \beta_i(t)$, where γ_k is the proportion of cases in Burkina Faso between 2002-2015 that observed in district k. As demonstrated by previous studies, regional climate variability impacts meningitis activities with epidemics occurring in the dry season and receding with the onset of the rainy season [4,5]. To capture the effect of seasonal changes on meningitis epidemics, we allow the transmission parameter $\beta_i(t)$ in Eq. (1) to vary over time according to:

$$\beta_i(t) = \begin{cases} \gamma_{i,1}\big(a_0 + a_{1,q}\cos 2\pi(t + a_2)\big), & \text{if } q \le t \le (2q+1)/2, \\ \gamma_{i,1} a_0, & \text{otherwise.} \end{cases} \tag{2}$$

In Eq. (2), the integer q denotes the year number of time t, and the condition $q \le t \le (2q+1)/2$ implies that this seasonality effect is present only during the first half of each year. This is consistent with historical data that show Meningitis epidemics occur in dry seasons and disappear with the onset of rainy seasons that last approximately 4 months from May/June to September (Fig 2A). Parameter a_0 in Eq. (3) is the baseline transmissibility which is not influenced by the seasonality effect, and the parameter $a_{1,q}$ represents the maximum magnitude of seasonality effect during year q. To account for between-year variation in transmission due to other external causes, we allow $a_{1,q}$ to be randomly drawn from a Uniform probability distribution. We found that this approach, which has been previously employed in other models[6], is important for the generation of epidemics with the ranges of magnitudes observed in

historical data (Fig 2A). The phase parameter a_2 is also included to provide additional flexibility in the modeling of the seasonality effect. We chose $a_2 = -0.25$ as it produces aseasonality effect that gradually increases from January, peaks around May and diminishes by September, consistent with the observed incidence time-series (Fig 2A).

As meningitis case incidence and carriage prevalence vary across age groups [7-10], we also allow the transmission parameter $\beta_i(t)$ to be age-dependent: in Eq. (3), parameter $\gamma_{i,1}$ denotes the relative force of infection in age group i with respect age group $\{< 1\}$ in the absence of seasonality. We estimate parameters $\gamma_{i,1}$'s, and $a_{1,q}$'s using the calibration procedure described below.

Calculating the Daily Contact Rates

In the absence of reliable between-district travel data, we use the following approach to model the contact patterns between individuals residing in different districts. Let $d_{k,k'}$ denote the distance between districts k and k', and n_k denote the population of district k. We use $\lambda_{(k,j)\leftarrow(k',j')}$ to denote the rate at which an average individual in age group i of district k contact with individuals in age group i' of district k'. Assuming that during each day an individual in age group i contacts on average with $\bar{\lambda}_{i,i'}$ individuals in age group i', we estimate $\lambda_{(k,j)\leftarrow(k',j')}$ by:

$$\lambda_{(k,j)\leftarrow(k',j')} = \bar{\lambda}_{i,i'} \frac{f(d_{k,k'})\frac{n_k}{n_{k'}}}{\sum_{k'} f(d_{k,k'})\frac{n_k}{n_{k'}}}, \tag{3}$$

where f is a monotonically decreasing function. Here, we use $f(d) = e^{-\alpha d}$, where the parameter $\alpha > 0$ is assumed to be constant over time and determines how the distance between two districts influences the intensity of contacts between them – higher values of α results in contacts that are more concentrated within districts. Eq. (3) holds the assumption that the number of contacts between two districts (k, k') is proportional to the multiple of some distance weight function (i.e. $f(d_{k,k'})$) and the relative size of the districts (i.e. $\frac{n_k}{n_{k'}}$). We employ the Haversine formula [11] and the coordinates of districts to approximate distance between districts (i.e. $d_{k,k'}$), and we estimate the daily contact rates among different age groups (i.e. $\bar{\lambda}_{i,i'}$) based on the results of a recent study that projects the Burkina Faso's social contact matrix [12]. Let $\hat{\lambda}_{j,j'}$ denote the average number of contact persons between age groups j and j' per day per survey participant as estimated by Prem et al. [12]. Age groups considered in this survey include $\hat{\mathcal{A}} =$ {0-4, 5-9, 10-14, 5-19, 20-24, 25-29, 30-34, 35-39, 40-44, 45-49, 50-54, 55-59, 60-64, 65-69, 70, 75+}.

Since our model only includes age groups $\mathcal{A} =$ {<1, 1-4, 5-14, 15-18, 19-29, 30+}, we first use this study estimate $\hat{\lambda}_{j,j'}$, $j, j' \in \hat{\mathcal{A}}$, to calculate the contact matrix $[\lambda_{,i,i'}]$, $i, i' \in \mathcal{A}$. To this end, we make the following assumption:

1. Contacts *within* an age group are uniformly distributed; that is, a contact with an average member in age group {0-4}, for example, is equally likely to be with someone of age 0, 1, 2, 3, or 4.

2. If in estimating $\lambda_{,i,i'}$, the age group $i' \in \mathcal{A}$ includes multiple age groups from $\hat{\mathcal{A}}$ (say $j'_1, j'_2, \ldots$), then $\lambda_{i,i'} = \sum_{j' \in \{j'_1, j'_2, \ldots\}} \hat{\lambda}_{j,j'}$. For example, $\lambda_{\{5-9\},\{30+\}} = \hat{\lambda}_{\{5-9\},\{30-34\}} + \hat{\lambda}_{\{5-9\},\{35-39\}} + \hat{\lambda}_{\{5-9\},\{40-44\}} + \hat{\lambda}_{\{5-9\},\{45-49\}} + \hat{\lambda}_{\{5-9\},\{50-54\}} + \cdots + \hat{\lambda}_{\{5-9\},\{70+\}}$.
3. If in estimating $\lambda_{i,i'}$, age group $i \in \mathcal{A}$ overlaps with multiple age groups from $\hat{\mathcal{A}}$ (say $i'_1, i'_2, \ldots$), the daily contact $\lambda_{i,i'}$ will be proportional to the population size of age groups $i'_1, i'_2, \ldots$. For example, $\lambda_{\{5-14\},\{30-34\}} = \frac{\varpi_{\{5-9\}}\lambda_{\{5-9\},\{30-34\}} + \varpi_{\{10-14\}}\lambda_{\{10-14\},\{30-34\}}}{\varpi_{\{5-9\}} + \varpi_{\{10-14\}}}$, where ϖ_i is the population of age group $i \in \hat{\mathcal{A}}$.

Following the approach of Medlock et al. [13], we then ensured that the number of contacts between age groups is symmetric (i.e. $\varpi_{i'}\lambda_{i,i'} = \varpi_{i,}\lambda_{i',i}$), by using $\bar{\lambda}_{i,i'} = \frac{1}{2\varpi_{i'}}(\varpi_{i'}\lambda_{i,i'} + \varpi_{i,}\lambda_{i',i})$. The contact rate matrix $[\bar{\lambda}_{i,i'}]$ used in our model is shown in Fig B.

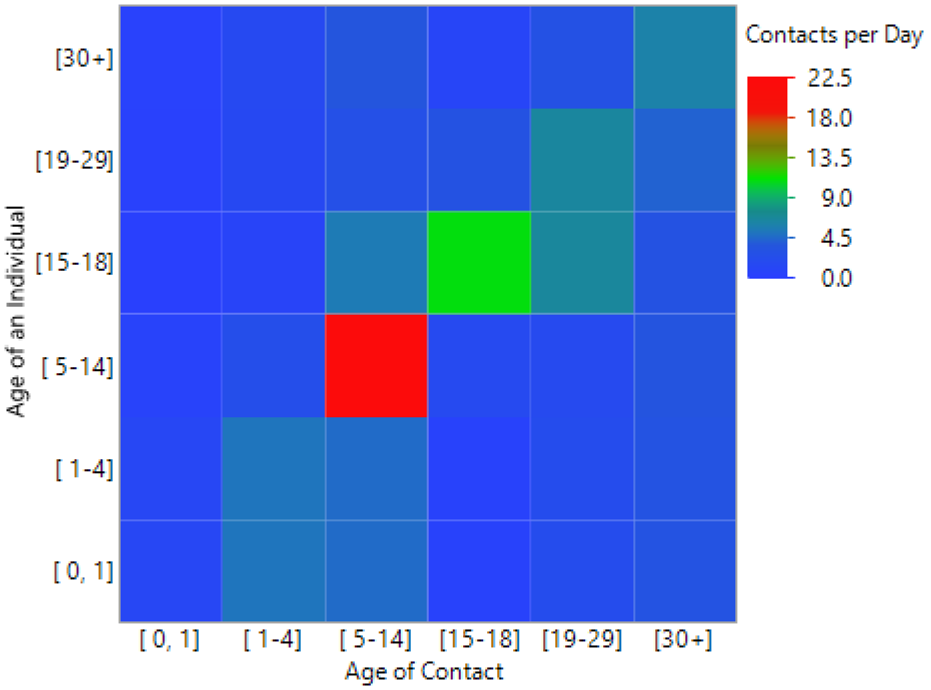

Fig B: Daily number of contacts that an average member of age groups on y-axis makes with members of age groups on x-axis. The asymmetry in this matrix results from the different sizes of the age groups.

S3. Calibration

The proposed calibration approach relies on the use of common random numbers [14,15] to simulate stochastic epidemic trajectories. This technique is often used to reduce variation when comparing between two or more alternative configurations by using the same vectors of uniform random variates while simulating alternatives. In this method, to obtain one simulated epidemic trajectory, we first specify the seed of the simulation's random number

generator (RNG) object. The simulator will then use the RNG object to generate a unique stream of random numbers which will be used to both draw a sample for epidemic parameters and to generate one simulated trajectory. This approach will enable us to regenerate any desired trajectory by providing the corresponding RNG seeds.

We use the approach described below to approximate the likelihood of observations given a simulated trajectory. Our chosen pseudolikelihood function consists of 4 components as explained below. In the following, we use $i \in \{1,2,\dots,5\}$ to denote the age groups {<1, 1-4, 5-14, 15-29, 30+} and $k \in \{1,2,\dots,55\}$ to denote districts of Burkina Faso.

Component 1. Likelihood of age-distribution of cases

Let $\hat{\tau}_i$ denote the number of meningococcal cases in age group $i \in \{1,2,\dots,5\}$ observed during 2007-2011 [16]: $\widehat{\mathrm{T}} = (\hat{\tau}_1, \hat{\tau}_2, \hat{\tau}_3, \hat{\tau}_4, \hat{\tau}_5) = (55, 224, 498, 152, 77)$. We assume that $(\hat{\tau}_1, \hat{\tau}_2, \hat{\tau}_3, \hat{\tau}_4, \hat{\tau}_5)$ follows a multinomial distribution with $\sum_{i=1}^{5} \hat{\tau}_i = 1006$ trials and success probabilities $\mathrm{T_z} = (\tau_{z,1}, \tau_{z,2}, \tau_{z,3}, \tau_{z,4}, \tau_{z,5})$, where $\tau_{z,i}$ is the percentage of meningococcal cases that belong to age group i in a simulated trajectory for which RNG seed z is used.

Component 2. Likelihood of average carriage prevalence

Let $\hat{S}_i$ and $\hat{s}_i$ be, respectively, number of participants and number of confirmed as meningococcal in age group $i \in \{1,2,\dots,5\}$ during the 2009-2012 meningococcal carriage survey study in the African meningitis belt [10]. We assume that $\hat{s}_i$ follows a binomial distribution with $\hat{S}_i$ trials and success probability $\rho_{z,i}$, where $\rho_{z,i}$ is the average prevalence of meningococcal carriage in age group i in a simulated trajectory for which RNG seed z is used, and $(\hat{s}_1, \hat{s}_2, \hat{s}_3, \hat{s}_4, \hat{s}_5) = (41, 228, 655, 450, 313)$ and $(\hat{S}_1, \hat{S}_2, \hat{S}_3, \hat{S}_4, \hat{S}_5) = (2199, 8839, 13121, 12425, 11906)$ [10].

Component 3. Likelihood of average weekly meningitis incidence

Let $\hat{y}_{k,t}$ denote the meningitis cases observed during week $t \in \{1,2,\dots,T\}$ from district $k \in \{1,2,\dots,K\}$, where $T = 728$ denotes the total number of weeks in 2002-2015 (data available in the accompanied Excel file). We assume that the mean of observed weekly meningitis cases from district $k \in \{1,2,\dots,K\}$, i.e. $\mu_{\hat{Y}_k} = \sum_{t=1}^{T} \hat{y}_{k,t} / T$, follows a student's t distribution with $T-1$ degrees of freedom and scaled to have mean of $\mu_{Y_{z,k}}$ and standard deviation of $\sigma_{Y_{z,k}}$, where $\mu_{Y_{z,k}}$ and $\sigma_{Y_{z,k}}$ are, respectively, the mean and standard deviation of weekly meningitis cases from district k in a simulated trajectory for which RNG seed z is used.

Component 4. Likelihood of periodicity in past meningitis epidemics

As described in a greater detail in §S4, to identify the significant periods at which past meningitis epidemics had occurred in Burkina Faso, we used the discrete Fourier transform. Let $\hat{\mathcal{F}}_k$ be the vector of Fourier amplitudes for the observed meningitis incidence time-series $\hat{Y}_k = (\hat{y}_{k,1}, \hat{y}_{k,2}, \dots, \hat{y}_{k,T})$ from district k (see the next section for details on how $\hat{\mathcal{F}}_k$ can be calculated and Fig E for an example). Let $\mathcal{F}_{z,k}$ be the vector of Fourier amplitude for the time-

series $Y_{z,k} = (y_{z,k,1}, y_{z,k,2}, \ldots, y_{z,k,T})$, the weekly meningitis cases in district k during a simulated trajectory for which RNG seed z is used. We measure the likelihood of the observed $\hat{\mathcal{F}}_k$ given $\mathcal{F}_{z,k}$ as the product of the likelihood of the angle between vectors $\hat{\mathcal{F}}_k$and $\mathcal{F}_{z,k}$ and the and the likelihood of the magnitude of vector $\hat{\mathcal{F}}_k$ (denoted by $\|\hat{\mathcal{F}}_k\|$) given $\mathcal{F}_{z,k}$:

1. The angle between vectors $\hat{\mathcal{F}}_k$and $\mathcal{F}_{z,k}$ is calculated as: $\hat{\theta}_{z,k} = \text{acos}(\frac{\mathcal{F}_{z,k}.\widehat{\mathcal{F}_k}}{\|\mathcal{F}_{z,k}\|.\|\widehat{\mathcal{F}_k}\|})$. Angle 0 implies a perfect match between the significant periods of two time-series, and hence we assume that $\hat{\theta}_{z,k}$ follows a truncated normal with minimum 0 and standard division $\sigma_{\hat{\theta}_{z,k}}$. We set $\hat{\theta}_{z,k} = 5.1$ which results in a truncated normal distribution with 95% of the distribution below 10 degree.
2. The magnitude of vector $\hat{\mathcal{F}}_k$, i.e. $\|\hat{\mathcal{F}}_k\|$, is always a positive number and hence, we choose a normal distribution with mean $\|\mathcal{F}_{z,k}\|$ and standard deviation of $0.1\|\mathcal{F}_{z,k}\|$, truncated to be bounded from below at 0, to represent the likelihood of $\|\hat{\mathcal{F}}_k\|$ given $\mathcal{F}_{z,k}$.

Total pseudolikelihood

To summarize, we calculate the natural logarithm of the likelihood of observations given a simulated trajectory as:

$$\ln \mathcal{L}(\hat{\mathrm{T}}; \mathrm{T_z}) + \frac{1}{5}\sum_{k=1}^{5} \ln \mathcal{L}(\hat{s}_i; \hat{S}_i, \rho_{z,i}) + \frac{1}{55}\sum_{k=1}^{55}\left[\ln \mathcal{L}(\mu_{\hat{Y}_k}; T-1, \mu_{Y_{z,k}}, \sigma_{Y_{z,k}}) + \ln \mathcal{L}(\hat{\mathcal{F}}_k; \mathcal{F}_{z,k})\right], \quad (4)$$

where $\mathcal{L}(\hat{\mathrm{T}}; \mathrm{T_z})$ is the pseudolikelihood of age-distribution of cases (Component 1 as explained above), $\mathcal{L}(\hat{s}_i; \hat{S}_i, \rho_{z,i})$ is the pseudolikelihood of carriage prevalence in age group i (Component 2), $\mathcal{L}(\mu_{\hat{Y}_k}; T-1, \mu_{Y_{z,k}}, \sigma_{Y_{z,k}})$ is the pseudolikelihood of average weekly incidence (Component 3), and $\mathcal{L}(\hat{\mathcal{F}}_k; \mathcal{F}_{z,k})$ is the pseudolikelihood of periodicity of epidemics (Component 4).

We note that Eq. (4) provides an approximation to the true likelihood function which could not be calculated for our model due to the sparsity of data and unobservable compartments. Also, Eq. (4) assumes that the four components of the pseudolikelihood function are independent, and assumption that is necessitated by lack of data to characterize the correlation between these four components (for example, while a non-zero correlation between incidence and carriage time-series is expected, time-series data needed to estimate this correlation are not available).

To build a set of trajectories to evaluate the performance of vaccine strategies in Table 1, we first simulate $N_0 = 100{,}000$ epidemic trajectories, each of which uses parameter values that are randomly drawn from the *prior* probability distribution of epidemic parameters listed in Table S1-Table S3. These prior distributions are mainly informed by estimates extracted from existing scientific literature. When such estimates are not available, we identified prior distributions by experimenting with the model ("hand-fitting") to ensure the model can produce simulated trajectories that are consistent with past observations.

To improve the efficiency of the calibration procedure, we terminate the simulation of a trajectory once any of the following conditions is met:

1. Weekly meningitis incidence in each district exceeds 2 times the maximum of historical meningitis incidence data in each district historical data.
2. Case-carrier ratio, defined as the number of cases during a week divided by carrier prevalence, falls out of the range [0, 0.025] [17].
3. Carriage prevalence in each district and within each age group falls out of the range [0%, 40%] [10,18-20].

To estimate α (the parameter of the distance function $f(\cdot)$ in Eq. (3)), we use the following approach. We note that α determines the degree of which contacts are concentrated within districts as opposed to across districts. For example, for our population, setting $\alpha = 0.104$ implies that 95% of daily contacts of an average individual happen with someone from the same district. We used the calibration procedure described above for $\alpha \in \{0.45, 0.056, 0.077, 0.104, 0.278\}$ which correspond to 70%, 80%, 90%, 95% and 99% of contracts being concentrated inside districts. Fig C demonstrates that the likelihood function (4) is not sensitive to the value of α and hence this parameter is not identifiable based on the available data. As such, we chose $\alpha = 0.077$ which is consistent with 90% of daily contacts of an average individual to occur within their home district; we have also tested the sensitivity of our findings to this assumption through a one-way sensitivity analysis (Fig J-Fig K).

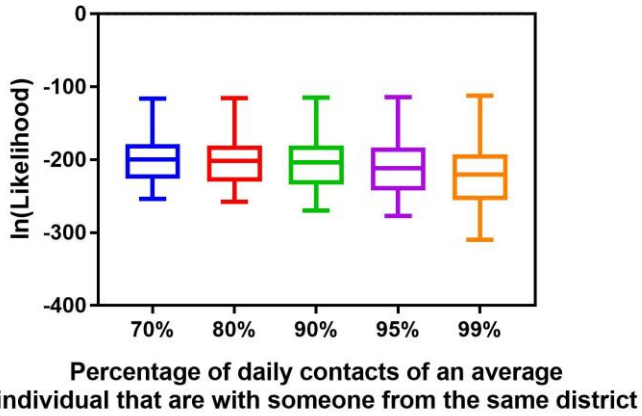

Fig C: Box plot of estimated likelihood (Eq. (4)) for 500 simulated trajectories with the highest likelihood value identified by the calibration procedure described in §S3 for different values of $\boldsymbol{\alpha}$. $\boldsymbol{\alpha} \in \{\mathbf{0.45}, \mathbf{0.056}, \mathbf{0.077}, \mathbf{0.104}, \mathbf{0.278}\}$ corresponds to 70%, 80%, 90%, 95% and 99% of contacts concentrated within districts.

Model Fit and Projections

Since PMC vaccines induce longer-term immunity, a full evaluation of the performance of our vaccination strategies requires us to run the model beyond the calibration period (which spans 14 years from 2002 to 2015). To extend these simulations, we assume that all model parameters determined through the calibration procedure described

below remain the same beyond 2015, but to allow the potential for the occurrence of sporadic epidemics, we assume that the external force of infection (represented by $a_{1,q}$ in Eq. (3)) repeats every 14 years. That is $a_{1,q+14} = a_{1,q}$ for $q \in \{1,2,3,\dots\}$.

Fig D and Fig 3F (in the main text) confirm the ability of the model to match the district-level epidemics in terms of (1) the average number of meningitis cases (Fig D) and (2) average number of districts in which the epidemic threshold of 10 meningitis cases per 100,000 population has been crossed (Fig 3F). The ability to capture district-level meningitis epidemics is critical for the accurate estimation of cost and health benefits of reactive campaigns.

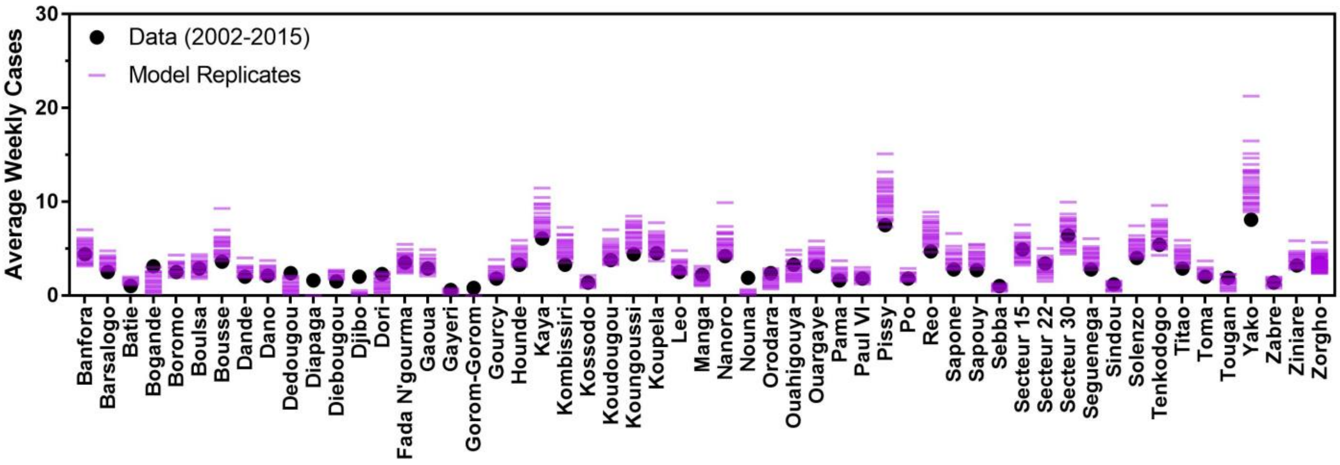

Fig D: Comparing the average weekly clinical meningitis cases observed between 2002-2015 in 55 districts of Burkina Faso (black circles) with those observed in 50 simulated trajectories (purple dashes).

Table S1: Prior distributions of natural history parameters

Parameter	Prior Distribution	Sources to Form Prior Distribution
Time until progression to disease from "Carrier Will Progress"	Uniform [1, 30] days	[21]
Time until losing carriage state in "Carrier Will Not Progress"	Uniform [7, 180] days	[3,22,23]
Time until losing immunity from carriage	Uniform [4, 260] weeks	[3,22,23]
Ratio of time until losing immunity from disease to time until losing immunity from carriage	Uniform [1, 20]	[3,22,23]
Probability of moving to "Carrier Will Progress" upon infection for age group <1 y	Uniform [0.5%, 3.0%]	[3,17]
Ratio of probability of moving to "Carrier Will Progress" upon infection with respect to age group <1 y		[3,17]
Age group 1-4 y	Uniform [0.175, 0.525]	
Age group 5-14 y	Uniform [0.09, 0.27]	
Age group 15-18 y	Uniform [0.085, 0.255]	
Age group 19-29 y	Uniform [0.085, 0.255]	
Age group 30+ y	Uniform [0.09, 0.27]	
Duration of disease	1 week	[24]
Disease-associated mortality	10%	[25]

Table S2: Prior distributions of transmission parameters (Eqs.(1)-(2))

Parameter	Prior Distribution	Sources to Form Prior Distribution
Transmission parameter for age group <1 y ($\gamma_{1,1}$)	Uniform [0, 0.000028]	Experiments with the model
Relative force of infection with respect to age group <1 y		Experiments with the model
Age group 1-4 y ($\gamma_{2,1}$)	Uniform [1.8, 2.2]	
Age group 5-14 y ($\gamma_{3,1}$)	Uniform [3.6, 4.4]	
Age group 15-18 y ($\gamma_{4,1}$)	Uniform [1.8, 2.2]	
Age group 19-29 y ($\gamma_{5,1}$)	Uniform [1.8, 2.2]	
Age group 30+ y ($\gamma_{6,1}$)	Uniform [0.9, 1.1]	
Seasonality parameters		Experiments with the model
a_0	1	
$a_{1,q}$ for year $q \in \{1,2,3,\dots\}$	Uniform [0, 400]	
a_2	-0.25	

Table S3: Prior distributions of the initial population structure on January 1st of the first year of the simulation warm-up period

Parameter	Prior Distribution	Sources to Form Prior Distribution
% of population in "Carrier Will Progress"	Uniform [0%, 0.5%]	Assumption
% of population in "Carrier Not Progress"	Uniform [0%, 10%]	[7,24]
% of population in "Immune from Disease"	Uniform [0%, 5%]	Assumption
% of population in "Immune from Carriage"	Uniform [0%, 80%]	[26]
% of population in "Meningitis"	0%	Assumption

Note: % of population in "Susceptible" is 1 minus the sum of samples drawn from the above probability distributions

S4. Identifying the Significant Periods of Past Epidemics

To identify the significant periods at which past meningitis epidemics had occurred in Burkina Faso, we used the discrete Fourier transform (DFT) of weekly meningitis cases over 2002-2015. The discrete Fourier transform of time-series $(f_0, f_1, \dots, f_n, \dots f_{N-1})$ is defined as:

$$F_k = \sum_{n=0}^{N-1} f_n e^{-2\pi ikn/N}, k \in \{0,1,\dots,N-1\},$$

where $e^{-2\pi ikn/N} = \cos\left(2\pi k \frac{n}{N}\right) - i \sin\left(2\pi k \frac{n}{N}\right), k \in \{0,1,\dots,N-1\}$, represent N Fourier bases with corresponding periods $\frac{N}{k} dt$ where dt is the time between two adjacent points in the time-series (here 1 week), and F_k represents projection of the time-series $(f_0, f_1, \dots, f_n, \dots f_{N-1})$ onto the corresponding Fourier bases. Since the meningitis incidence time-series is real-valued, $F_k, k \in \{0,1,\dots,\lfloor N/2 \rfloor\}$ represents all the periodicity of the signal with F_0 representing zero frequency (or simply the sum of the time-series) and $F_{\lfloor n/2 \rfloor}$ representing maximum possible frequency (Note: $\lfloor N/2 \rfloor = N/2$ if N is an even number and $\lfloor N/2 \rfloor = (N-1)/2$ if N is an odd number. For a comprehensive review of Fourier transform, refer to the tutorial by Duhamel and Vetterli [27]. $|F_k|^2$ is referred to as Fourier amplitude and we use $\mathcal{F} = (|F_0|^2, |F_1|^2, \dots, |F_{\lfloor N/2 \rfloor}|^2)$ to denote the vector of Fourier amplitudes.

To identify significant periods of a time-series (i.e. periods for which $|F_k|^2$ is statistically greater than zero) we generate 1,000 bootstrap resamples of the time-series by randomly shuffling the time-points of the original time-series [28]. This process removes the inherited periodicity in each resampled time-series. We next use DFT to calculate the Fourier weights $|F_k|^2, k \in \{0,1,\dots,N-1\}$, for each bootstrap resampled time-series. We consider the period that correspond to the Fourier weight $|F_k|^2$ statistically significant, if $|F_k|^2$ calculated for the original time-series is greater than 99% of the $|F_k|^2$ calculated from the 1,000 bootstrap samples. Panel B in Fig E displays the significant periods of weekly meningitis cases between 2002-2015 identified using the approached described above.

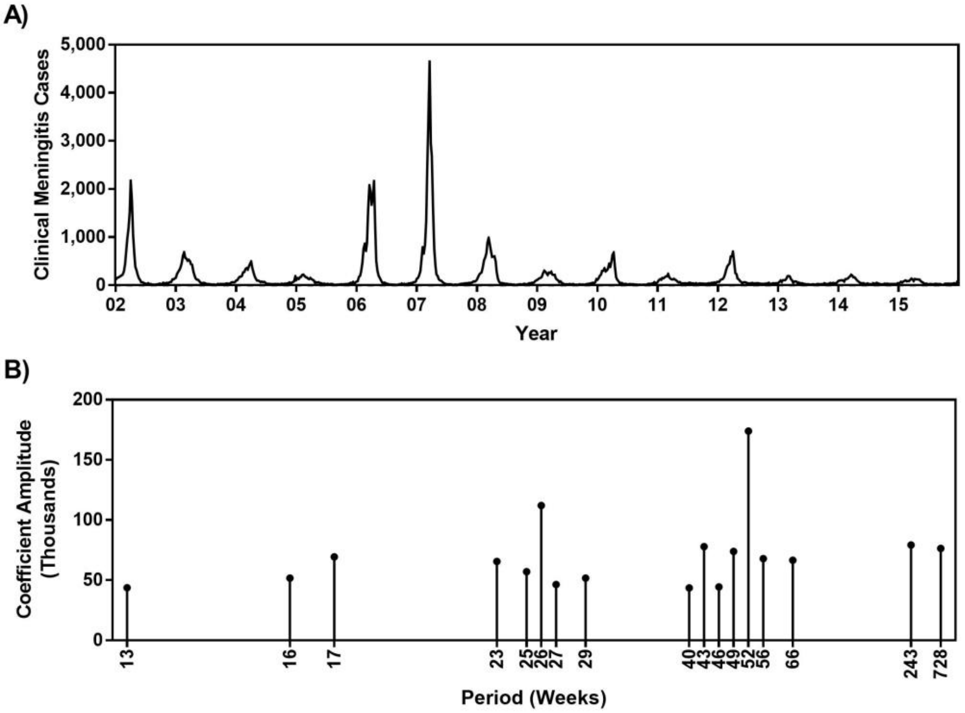

Fig E: A) Weekly meningitis cases in Burkina Faso reported between 2002-2015. B) Periods with significant contribution (x-axis) and level of each period contribution (y-axis) to the occurrence of past meningitis epidemics between 20020-2015 displayed in A. The periods with significant Fourier coefficient amplitude are primarily clustered around weeks 23-29, 40-66 signaling strong biannual and annual signals in data. See §S4 for the description of the approach used to identify these significant periods.

S5. Estimating the Meningococcal Incidence Time-Series

Since reactive vaccination campaigns are triggered at the district level once the number of clinical meningitis cases has passed the WHO epidemic threshold,[29,30] it is essential that the model be able to replicate observed temporal patterns of incidence of clinical meningitis cases (Fig 2A). The clinical meningitis incidence time-series, however, cannot be used to estimate the cost and health outcomes of meningococcal vaccination strategies because clinical meningitis data also include cases caused by other pathogens such as *Streptococcus pneumoniae* and *Haemophilus influenzae* type b (Fig 2B) and meningococcal vaccines are not expected to directly impact the carriage prevalence and disease due to these other pathogens. As such, we use the following approach to estimate the meningococcal incidence and mortality from case series data, which can then be used to measure the costs and health outcomes of each meningococcal vaccination strategies.

Let μ be the average percentage of clinical meningitis cases attributable to *N. meningitidis* in past meningitis epidemics. Using the data presented in Fig 2, we estimate $\mu = 70.8\%$ and $\mu = 37.9\%$ for scenarios with and without strain-replacement, respectively. Let $I_{z,i}$ and $\rho_{z,i}$ be, respectively, the total meningococcal cases and average

meningococcal carriage prevalence in age group i in a simulated trajectory for which RNG seed z is used and no additional vaccination interventions other than routine immunization with MenAfriVac have been employed. If this simulation trajectory lasts for T years, $\frac{\mu I_{z,i}}{T\rho_{z,i}}$ is the meningococcal case-carriage ratio for age group i.

Let $\rho'_{z,i}(t)$ be the meningococcal carriage prevalence at the simulation time t in age group i when the vaccination strategy of interest (Base, Base Prime, Elimination 1, or Elimination 2 as described in Table 1) is in effect. For this simulated trajectory, we estimate the meningococcal weekly cases among age group i by $\frac{\mu I_{z,i}}{T\rho_{z,i}}\rho'_{z,i}(t)$. We use these estimated meningococcal incidence and mortality time-series to calculate the cost of case management and the disability-adjusted life-years due to meningococcal epidemics under each vaccination strategies described below.

S6. Cost and DALY Calculation

We use disability-adjusted life-years (DALY) to measure the health outcome associated with each vaccine strategy. We assume that on average 7.2% (4.3-11.2%) of those surviving meningitis episode will experience a major sequela that increases annual disability weight to 0.21 (0.139 - 0.298) [31]. We estimate the cost incurred from vaccination strategies and costs averted due to the prevention of meningitis cases from the perspective of the payer. We chose this perspective because the cost of vaccination programs in Burkina Faso is borne primarily by the government and donors, and hence, the demonstration of affordability is essential for these programs to be considered in practice. Our model accounts for costs incurred due to meningitis case management, short term care for patients who experience sequelae, and the operation costs of routine, reactive, and preventive vaccination campaigns. Both costs and health outcomes are discounted at an annual rate of 3%. All costs are presented in the 2016 US dollars. If the cost of an item was estimated in a year prior to 2016, we used the inflation rate of 2% to calculate the equivalent monetary value in 2016 [32].

Guided by a recent cost study in Burkina Faso,[33] we use US $50.73 (estimated in 2015, $51.74 in 2016) as an estimate for the costs of meningitis case management for the health system. This estimate includes the costs of prepositioning and distribution of medicines during epidemics, district laboratory analyses for case diagnosis and choice of treatment, and patient care. During the 2006-2007 meningitis season in Burkina Faso, household in which a person experienced sequelae incurred an additional cost of US $25.4-$154.4 for rehabilitation [34]. We assumed that short-term cost of sequelae is $107.29 in 2016.

MenAfriVac is expected to be added to Expanded Program for Immunization by year 2020. We therefore consider the capital costs (i.e. buildings, vehicles, and cold chain equipment) of routine vaccination programs that will be implemented after 2020 and use MenAfriVac or other meningococcal conjugate vaccine to be sunk costs. Likewise, during reactive and preventive campaigns, capital costs are expected to be negligible since the required infrastructure and equipment are most likely available through the Expanded Program for Immunization [35]. As such, recurring

costs are assumed to constitute the vast majority of the routine, reactive and preventive vaccination campaign costs [33,35,36]. We used the delivery cost of $0.28 (estimated in 2010, $0.31 in 2016) per dose for routine vaccination programs that include service delivery, advocacy and communication, monitoring and disease surveillance, program management [33]. To calculate the total vaccine doses required for routine immunization, we assumed a wastage factor of 1.67 [33]. For both reactive and preventive vaccination campaigns, we assumed that the vaccine delivery cost (which includes transportation, advocacy and communication, personnel, monitoring and disease surveillance, cold chain equipment, and program management) is US $0.43 (estimated in 2007, $0.51 in 2016) per person[35].

Vaccine and injection supplies are major contributors to recurring costs during reactive and preventive campaigns [35]. We assume $0.15 for injection supplies and $0.49 per MenAfriVac dose and $4 per polyvalent meningococcal polysaccharide vaccine (PMPV) dose [37]. As the price of polyvalent meningococcal conjugate vaccine (PMCV) is not yet determined, we vary this price from $2 to $10 per dose.

The summary of cost parameters is provided in Table S4.

Table S4. Cost parameters (represented in 2016 US$)

Parameters	Cost	Sources/Notes
**Meningitis**		
Case management	$51.74	[33]
Sequelae	$107.29	On average, 7.2% of those surviving a meningitis episode will experience major sequela. [34]
**Vaccine prices**		
MenAfriVac	$0.49 per dose	[37]
Polyvalent meningococcal polysaccharide vaccine (PMPV)	$4.0 per dose	[37] -- varied between $2.0 and $4.0 in a sensitivity analysis.
Polyvalent meningococcal conjugate vaccine (PMCV)	$4.0 per dose	Price is not determined yet; varied between $4.0 and $10.0 in a sensitivity analysis
**Vaccine delivery costs**		
Routine immunization programs	$0.52 per vaccinated person	The delivery cost is estimated $0.31 per dose for routine vaccination programs[33] and we used a wastage factor of 1.67[33] to estimate delivery cost per vaccinated person ($0.31/1.67 = $0.52).
Reactive immunization campaigns	$0.51 per vaccinated person	[35]
Preventive immunization campaigns	$0.51 per vaccinated person	[35]
**Other vaccination costs**		
Injection supply	$0.15 per vaccinated person	[26]

S7. Reactive Campaign Response Time

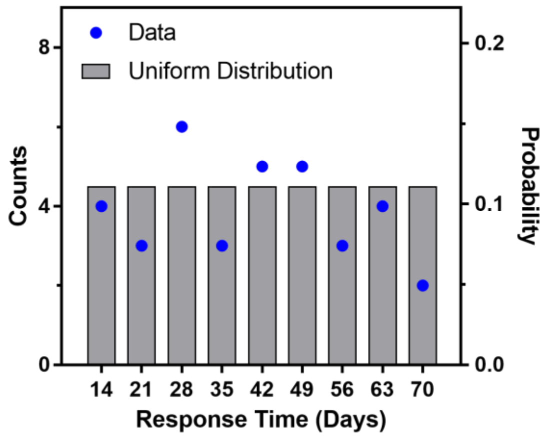

Fig F: Distribution of reactive campaign response time (days from reaching the epidemic threshold to vaccine implementation; data obtained from Trotter et al. [38] for serogroup W epidemics).

S8. Long-Term Impact of Vaccination Strategies

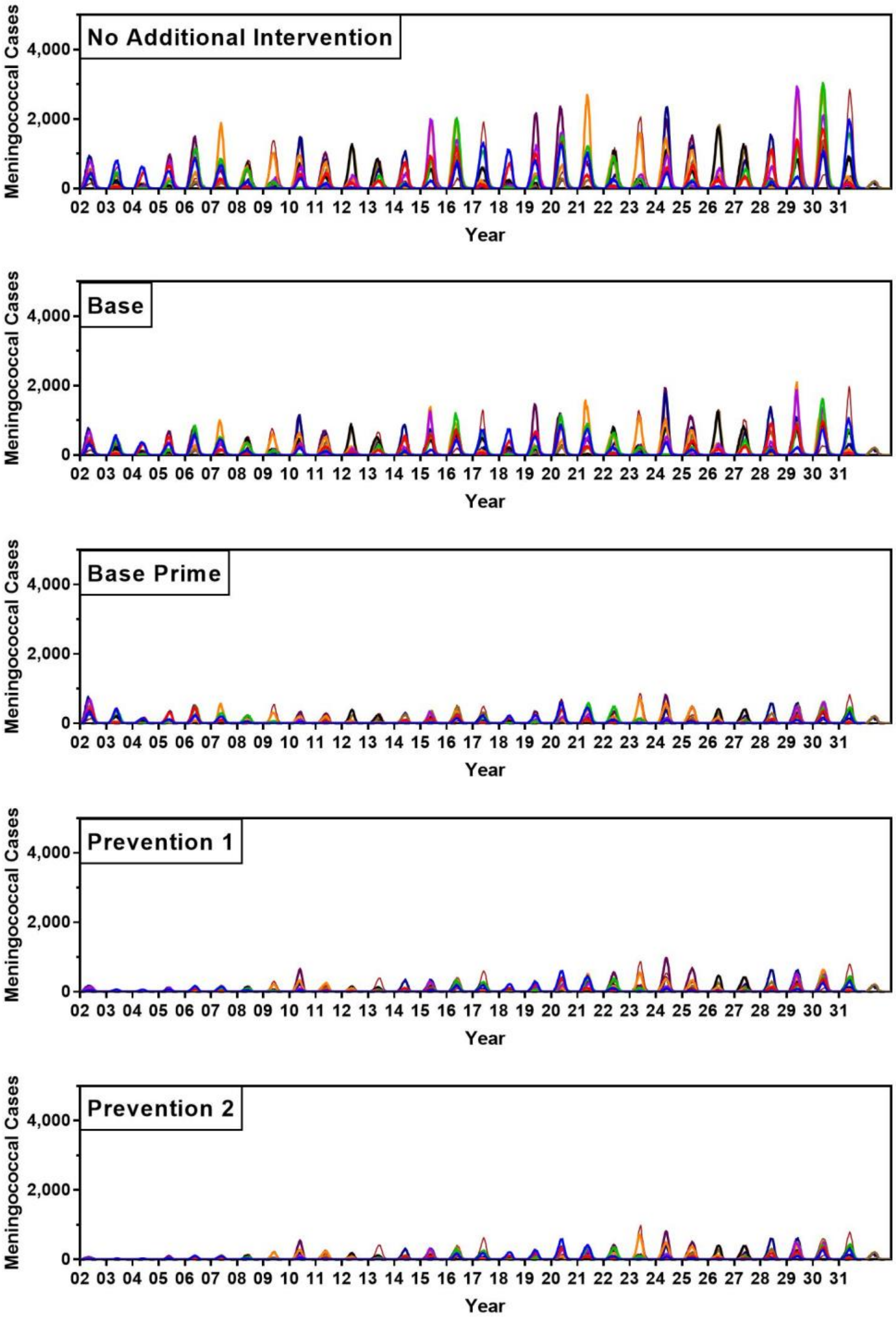

Fig G: Comparing the long-term impact of different vaccination strategies for 10 typical simulations. Under strategies that utilize PMC vaccines (Base Prime, Prevention 1 and Prevention 2), meningitis outbreaks may recur 10-15 years after the implementation of the first mass preventive campaign when the immunity induced by PMC vaccines begins to wane in the adult population. The “No Additional Intervention” strategy (top figure) represents a counterfactual scenario where reactive vaccination campaigns are never used. Each colored curve in these figures represents a simulated trajectory.

S9. Impact of Vaccine Prices on the Performance of Vaccination Strategies

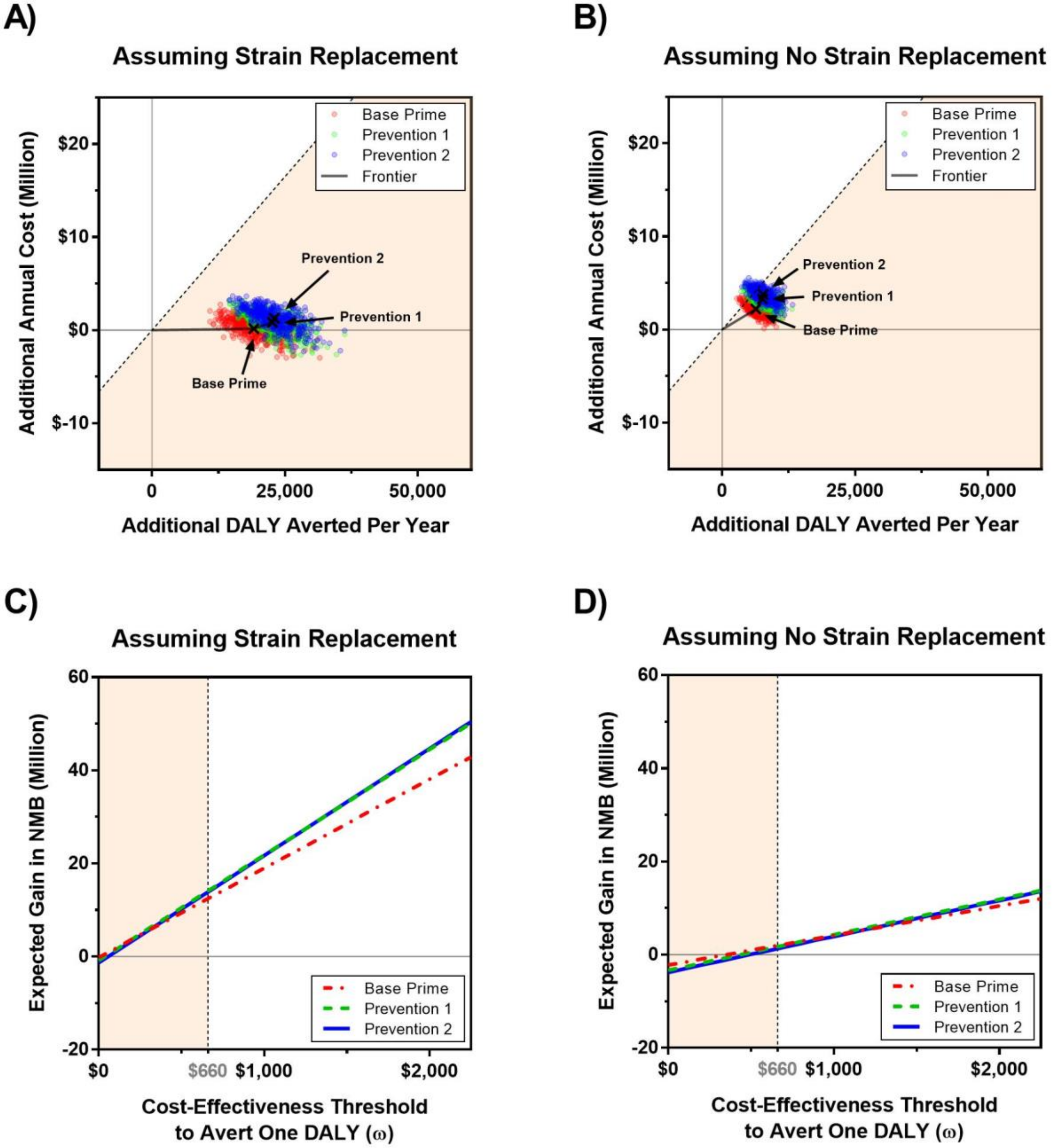

Fig H: Economic evaluation of vaccine strategies described in Table 1 for scenarios with and without strain replacement where the price of PMP and PMC vaccines are $2 and $4 per dose, respectively. In cost-effectiveness planes A and B, each dot represents the additional cost and disability-adjusted life-years (DALY) averted in a simulated trajectory with respect to the Base strategy which represents the current WHO policy that relies on reactive vaccination campaigns using PMP vaccines in districts where the epidemic threshold is passed. The x's represent the expected additional cost and DALYs averted with respect to the Base strategy. Figs C and D show the expected gain in net monetary benefit (NMB) of a strategy with respect to the Base strategy for a given cost-effectiveness threshold ω. The diagonal dashed line in Figs A and B and the vertical dashed line in Figs C and D represents the cost-effectiveness threshold of one per capita gross domestic product of Burkina Faso which is estimated to be 660 USD in 2015 [48]. All costs and DALYs are discounted at rate 3% to year 2016.

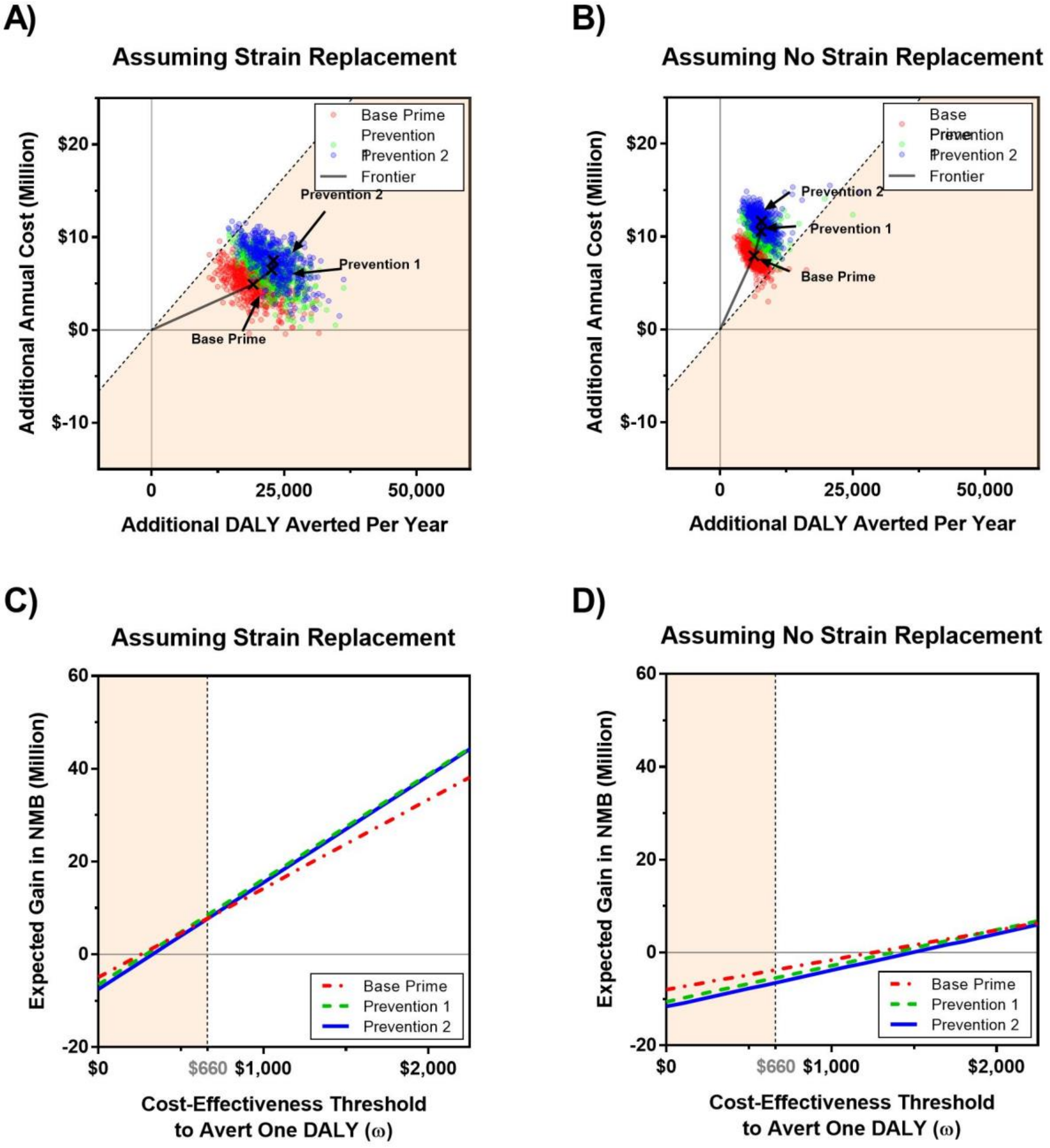

Fig I: Economic evaluation of vaccine strategies described in Table 1 for scenarios with and without strain replacement where the price of PMP and PMC vaccines are $4 and $10 per dose, respectively. In cost-effectiveness planes A and B, each dot represents the additional cost and disability-adjusted life-years (DALY) averted in a simulated trajectory with respect to the Base strategy which represents the current WHO policy that relies on reactive vaccination campaigns using PMP vaccines in districts where the epidemic threshold is passed. The x's represent the expected additional cost and DALYs averted with respect to the Base strategy. Figs C and D show the expected gain in net monetary benefit (NMB) of a strategy with respect to the Base strategy for a given cost-effectiveness threshold ω. The diagonal dashed line in Figs A and B and the vertical dashed line in Figs C and D represents the cost-effectiveness threshold of one per capita gross domestic product of Burkina Faso which is estimated to be 660 USD in 2015 [48]. All costs and DALYs are discounted at rate 3% to year 2016.

S10. Impact of Distance Parameter α on the Performance of Vaccination Strategies

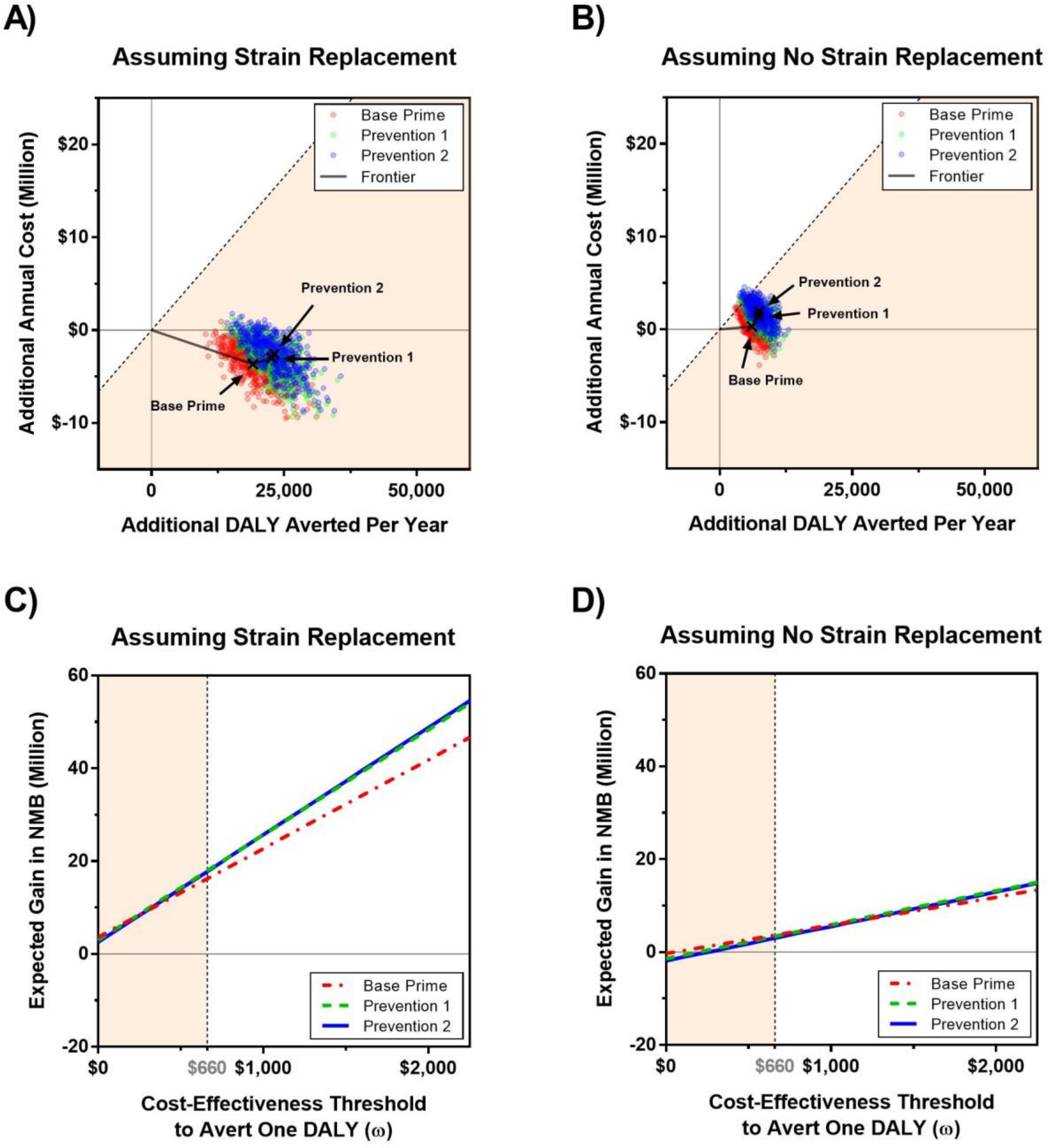

Fig J: Economic evaluation of vaccine strategies described in Table 1 for scenarios with and without strain replacement where the price of PMP and PMC vaccines are $4 per dose, respectively, and $\alpha = 0.056$ (i.e. 80% of contacts are concentrated within districts)**.**

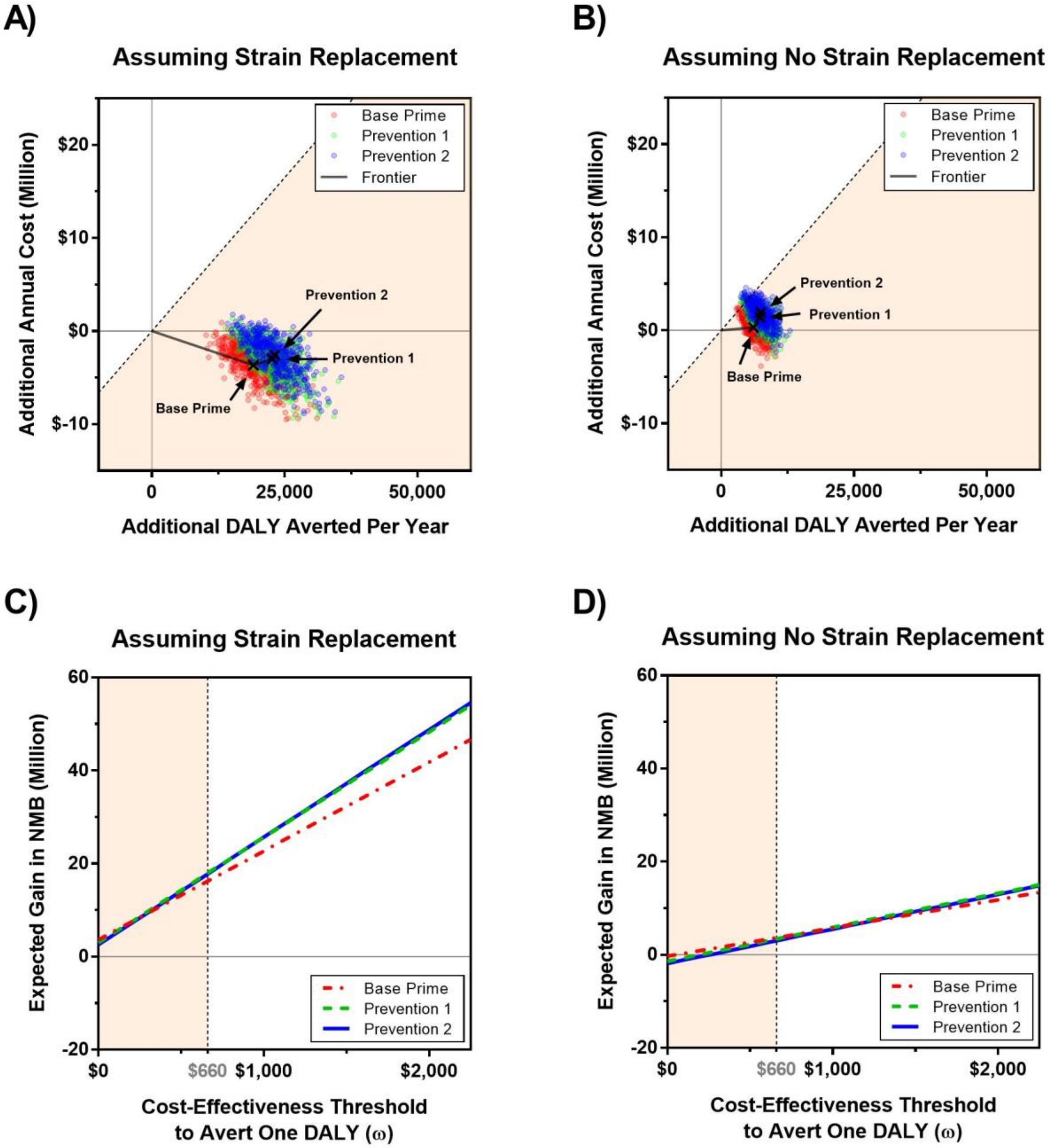

Fig K: Economic evaluation of vaccine strategies described in Table 1 for scenarios with and without strain replacement where the price of PMP and PMC vaccines are \$4 per dose, respectively, and $\alpha = 0.104$ (i.e. 95% of contacts are concentrated within districts)**.**

S11. CHEERS Guideline for Reporting Health Economic Evaluations

We followed the Consolidated Health Economic Evaluation Reporting Standards (CHEERS)[39] to report the results of our cost-effectiveness analysis study. CHEERS is developed by ISPOR Health Economic Evaluation Publication Guidelines Good Reporting Practices Task Force with the objective of optimizing the reporting of health economic evaluation studies. The task force recommends including 24 items when reporting economic evaluations of health interventions.

References

1. Central Intelligence Agency. The World Factbook: Central Intelligence Agency; 2015 [August 4, 2016)]. Available from: https://www.cia.gov/library/publications/the-world-factbook/geos/uv.html.
2. World Health Organization. Life Tables by Country - Burkina Faso [updated September 15, 2015]. Available from: http://apps.who.int/gho/data/view.main.LT61990?lang=en.
3. Irving TJ, Blyuss KB, Colijn C, Trotter CL. Modelling meningococcal meningitis in the African meningitis belt. Epidemiol Infect. 2012;140(5):897-905. doi: 10.1017/S0950268811001385. PubMed PMID: 21781369.
4. Sultan B, Labadi K, Guegan JF, Janicot S. Climate drives the meningitis epidemics onset in west Africa. PLoS Med. 2005;2(1):e6. doi: 10.1371/journal.pmed.0020006. PubMed PMID: 15696216; PubMed Central PMCID: PMCPMC545199.
5. Agier L, Deroubaix A, Martiny N, Yaka P, Djibo A, Broutin H. Seasonality of meningitis in Africa and climate forcing: aerosols stand out. J R Soc Interface. 2013;10(79):20120814. doi: 10.1098/rsif.2012.0814. PubMed PMID: 23221989; PubMed Central PMCID: PMCPMC3565698.
6. Karachaliou A, Conlan AJ, Preziosi MP, Trotter CL. Modeling Long-term Vaccination Strategies With MenAfriVac in the African Meningitis Belt. Clin Infect Dis. 2015;61 Suppl 5:S594-600. doi: 10.1093/cid/civ508. PubMed PMID: 26553693; PubMed Central PMCID: PMCPMC4639487.
7. Christensen H, May M, Bowen L, Hickman M, Trotter CL. Meningococcal carriage by age: a systematic review and meta-analysis. Lancet Infect Dis. 2010;10(12):853-61. doi: 10.1016/S1473-3099(10)70251-6. PubMed PMID: 21075057.
8. Campagne G, Schuchat A, Djibo S, Ousseini A, Cisse L, Chippaux JP. Epidemiology of bacterial meningitis in Niamey, Niger, 1981-96. Bull World Health Organ. 1999;77(6):499-508. PubMed PMID: 10427935; PubMed Central PMCID: PMCPMC2557688.
9. Collard JM, Issaka B, Zaneidou M, Hugonnet S, Nicolas P, Taha MK, et al. Epidemiological changes in meningococcal meningitis in Niger from 2008 to 2011 and the impact of vaccination. BMC Infect Dis. 2013;13:576. doi: 10.1186/1471-2334-13-576. PubMed PMID: 24313998; PubMed Central PMCID: PMCPMC4029580.
10. MenAfriCar c. The Diversity of Meningococcal Carriage Across the African Meningitis Belt and the Impact of Vaccination With a Group A Meningococcal Conjugate Vaccine. J Infect Dis. 2015;212(8):1298-307. doi: 10.1093/infdis/jiv211. PubMed PMID: 25858956; PubMed Central PMCID: PMCPMC4577048.
11. Van Brummelen G. Heavenly mathematics: The forgotten art of spherical trigonometry: Princeton University Press; 2013.
12. Prem K, Cook AR, Jit M. Projecting social contact matrices in 152 countries using contact surveys and demographic data. Plos Computational Biology. 2017;13(9). doi: ARTN e1005697

10.1371/journal.pcbi.1005697. PubMed PMID: WOS:000411981000011.
13. Medlock J, Galvani AP. Optimizing influenza vaccine distribution. Science. 2009;325(5948):1705-8. Epub 2009/08/22. doi: 10.1126/science.1175570. PubMed PMID: 19696313.
14. Nelson BL, Matejcik FJ. Using common random numbers for indifference-zone selection and multiple comparisons in simulation. Management Science. 1995;41(12):1935-45.

15. Clark GM, editor Use of common random numbers in comparing alternatives. Proceedings of the 22nd conference on Winter simulation; 1990: IEEE Press.
16. Novak RT, Kambou JL, Diomande FV, Tarbangdo TF, Ouedraogo-Traore R, Sangare L, et al. Serogroup A meningococcal conjugate vaccination in Burkina Faso: analysis of national surveillance data. Lancet Infect Dis. 2012;12(10):757-64. doi: 10.1016/S1473-3099(12)70168-8. PubMed PMID: 22818241.
17. Koutangni T, Boubacar Mainassara H, Mueller JE. Incidence, carriage and case-carrier ratios for meningococcal meningitis in the African meningitis belt: a systematic review and meta-analysis. PLoS One. 2015;10(2):e0116725. doi: 10.1371/journal.pone.0116725. PubMed PMID: 25658307; PubMed Central PMCID: PMCPMC4319942.
18. Mueller JE, Yaro S, Njanpop-Lafourcade BM, Drabo A, Idohou RS, Kroman SS, et al. Study of a localized meningococcal meningitis epidemic in Burkina Faso: incidence, carriage, and immunity. J Infect Dis. 2011;204(11):1787-95. doi: 10.1093/infdis/jir623. PubMed PMID: 21998478; PubMed Central PMCID: PMCPMC3247801.
19. Kristiansen PA, Diomande F, Wei SC, Ouedraogo R, Sangare L, Sanou I, et al. Baseline meningococcal carriage in Burkina Faso before the introduction of a meningococcal serogroup A conjugate vaccine. Clin Vaccine Immunol. 2011;18(3):435-43. doi: 10.1128/CVI.00479-10. PubMed PMID: 21228139; PubMed Central PMCID: PMCPMC3067389.
20. Trotter CL, Greenwood BM. Meningococcal carriage in the African meningitis belt. Lancet Infect Dis. 2007;7(12):797-803. doi: 10.1016/S1473-3099(07)70288-8. PubMed PMID: 18045562.
21. Tzeng YL, Stephens DS. Epidemiology and pathogenesis of Neisseria meningitidis. Microbes Infect. 2000;2(6):687-700. PubMed PMID: 10884620.
22. Blakebrough IS, Greenwood BM, Whittle HC, Bradley AK, Gilles HM. The epidemiology of infections due to Neisseria meningitidis and Neisseria lactamica in a northern Nigerian community. J Infect Dis. 1982;146(5):626-37. PubMed PMID: 7130749.
23. Trotter CL, Gay NJ, Edmunds WJ. The natural history of meningococcal carriage and disease. Epidemiol Infect. 2006;134(3):556-66. doi: 10.1017/S0950268805005339. PubMed PMID: 16238823; PubMed Central PMCID: PMCPMC2870423.
24. Stephens DS, Greenwood B, Brandtzaeg P. Epidemic meningitis, meningococcaemia, and Neisseria meningitidis. Lancet. 2007;369(9580):2196-210. doi: 10.1016/S0140-6736(07)61016-2. PubMed PMID: 17604802.
25. World Health Organization. Control of epidemic meningococcal disease: WHO practical guidelines. Report No.: WHO/EMC/BAC/98.3. 1998.
26. Bovier PA, Wyss K, Au HJ. A cost-effectiveness analysis of vaccination strategies against N. meningitidis meningitis in sub-Saharan African countries. Soc Sci Med. 1999;48(9):1205-20. PubMed PMID: 10220020.
27. Duhamel P, Vetterli M. Fast Fourier-Transforms - a Tutorial Review and a State-of-the-Art. Signal Process. 1990;19(4):259-99. doi: Doi 10.1016/0165-1684(90)90158-U. PubMed PMID: WOS:A1990CZ36300001.
28. Wehrens R, Putter H, Buydens LMC. The bootstrap: a tutorial. Chemometr Intell Lab. 2000;54(1):35-52. doi: Doi 10.1016/S0169-7439(00)00102-7. PubMed PMID: WOS:000165553700003.
29. World Health Organization. Meningitis Outbreak Response in Sub-Saharan Africa: WHO Guideline, Report No. WHO/HSE/PED/CED/14.5. Geneva: 2014.
30. World Health Organization. Detecting meningococcal meningitis epidemics in highly-endemic African countries. Weekly Epidemiology Record (WER). Weekly Epidemiology Record. 2000;38:306–9.
31. World Health Organization. WHO methods and data sources for global burden of disease estimates 2000-2011. 2003.
32. Statista. Burkina Faso: Inflation rate from 2010 to 2020 (compared to the previous year) [September 16, 2016)]. Available from: http://www.statista.com/statistics/449025/inflation-rate-in-burkina-faso/.
33. Colombini A, Trotter C, Madrid Y, Karachaliou A, Preziosi MP. Costs of Neisseria meningitidis Group A Disease and Economic Impact of Vaccination in Burkina Faso. Clin Infect Dis. 2015;61 Suppl 5:S473-82. doi: 10.1093/cid/civ600. PubMed PMID: 26553677.
34. Colombini A, Bationo F, Zongo S, Ouattara F, Badolo O, Jaillard P, et al. Costs for households and community perception of meningitis epidemics in burkina faso. Clin Infect Dis. 2009;49(10):1520-5. doi: 10.1086/644623. PubMed PMID: 19842972.

35. Colombini A, Badolo O, Gessner BD, Jaillard P, Seini E, Da Silva A. Costs and impact of meningitis epidemics for the public health system in Burkina Faso. Vaccine. 2011;29(33):5474-80. doi: 10.1016/j.vaccine.2011.05.058. PubMed PMID: 21641952.
36. Le Gargasson JB, Nyonator FK, Adibo M, Gessner BD, Colombini A. Costs of routine immunization and the introduction of new and underutilized vaccines in Ghana. Vaccine. 2015;33 Suppl 1:A40-6. doi: 10.1016/j.vaccine.2014.12.081. PubMed PMID: 25919173.
37. UNICEF Vaccine Price Data [December 14, 2017]. Available from: http://www.unicef.org/supply/index_57476.html.
38. Trotter CL, Cibrelus L, Fernandez K, Lingani C, Ronveaux O, Stuart JM. Response thresholds for epidemic meningitis in sub-Saharan Africa following the introduction of MenAfriVac((R)). Vaccine. 2015;33(46):6212-7. doi: 10.1016/j.vaccine.2015.09.107. PubMed PMID: 26463444.
39. Husereau D, Drummond M, Petrou S, Carswell C, Moher D, Greenberg D, et al. Consolidated Health Economic Evaluation Reporting Standards (CHEERS)--explanation and elaboration: a report of the ISPOR Health Economic Evaluation Publication Guidelines Good Reporting Practices Task Force. Value Health. 2013;16(2):231-50. doi: 10.1016/j.jval.2013.02.002. PubMed PMID: 23538175.
